# Pax1a-EphrinB2a pathway in the first pharyngeal pouch controls hyomandibular plate formation by promoting chondrocyte formation in zebrafish

**DOI:** 10.3389/fcell.2025.1482906

**Published:** 2025-03-05

**Authors:** Haewon Jeon, Sil Jin, Jihyeon Kim, Saehoon Joo, Chong Pyo Choe

**Affiliations:** ^1^ Division of Applied Life Science, Gyeongsang National University, Jinju, Republic of Korea; ^2^ Division of Life Science, Gyeongsang National University, Jinju, Republic of Korea; ^3^ Plant Molecular Biology and Biotechnology Research Center, Gyeongsang National University, Jinju, Republic of Korea

**Keywords:** *pax1*, *ephrinb2*, hyomandibular plate, facial cartilages, zebrafish

## Abstract

The hyomandibular (HM) cartilage securing the lower jaw to the neurocranium in fish is a craniofacial skeletal element whose shape and function have changed dramatically in vertebrate evolution, yet the genetic mechanisms shaping this cartilage are less understood. Using mutants and rescue experiments in zebrafish, we reveal a previously unappreciated role of Pax1a in the anterior HM plate formation through EphrinB2a. During craniofacial development, *pax1a* is expressed in the pharyngeal endoderm from the pharyngeal segmentation stage to chondrocyte formation. Loss of *pax1a* leads to defects in the first pouch and to the absence of chondrocytes in the anterior region of the HM plate caused by increased cell death in differentiating osteochondral progenitors. In *pax1* mutants, a forced expression of *pax1a* by the heat shock before pouch formation rescues the defects in the first pouch and HM plate together, whereas a forced expression of *pax1a* after pouch formation rescues only the defects in the HM plate without rescuing the first pouch defects. In *pax1a* mutants, *ephrinb2a* expressed in the first pouch is downregulated when adjacent osteochondral progenitors differentiate into the chondrocytes, with mutations in *ephrinb2a* causing hyomandibular plate defects. Lastly, *pax1* mutants rescue the anterior hyomandibular plate defects by pouch-specific restoration of EphrinB2a or a heat-shock-treated expression of *ephrinb2a* after pouch formation. We propose that the Pax1a-EphrinB2a pathway in the first pouch is directly required to shape the HM plate in addition to the early role of Pax1a in the first pouch formation.

## Introduction

The shape change of the craniofacial skeleton is a driving force of the head shape change during vertebrate evolution. The hyomandibular (HM) cartilage is presented in the hyoid regions of fish, with its role in securing the lower jaw to the neurocranium (Medeiros and Crump, 2012; Iwasaki et al., 2022). This cartilage has undergone significant transformations in shape across various vertebrate species. In cartilaginous and basal bony fish, the HM cartilage is rod-shaped, while in advanced bony fish, it becomes plate-like (Edgeworth, 1926; Medeiros and Crump, 2012). In tetrapods, it evolved into the stapes, a tiny bone in the middle ear that is much smaller than the HM cartilage and functions to transmit sound (Takechi and Kuratani, 2010). While understanding the formation of HM cartilage and stapes could provide important insights into head evolution in vertebrates, the developmental mechanisms forming these skeletal elements have only recently been elucidated in fish and mice.

In zebrafish, HM cartilage arises in the dorsal region of the hyoid arch. At 36 h post-fertilization (hpf), a group of mesenchymal cells derived from the cranial neural crest aggregates and differentiates into osteochondral progenitors (Crump et al., 2004b; Sperber and Dawid, 2008; Barske et al., 2016). By 48 hpf, they proliferate and further differentiate into *sox9a*-expressing chondrocytes, contributing to HM cartilage (Eames et al., 2004; Flores et al., 2006). The aggregation and proliferation of osteochondral progenitors require Barx1, which marks these progenitors in all pharyngeal arches, with a loss-of-function mutation in *barx1* resulting in facial skeletal defects in zebrafish (Nichols et al., 2013). In the hyoid arch, *barx1* expression is regulated by positive Endothelin1 and inhibitory Jagged-Notch signals from adjacent head ectoderm and cranial neural crest-derived mesenchyme, respectively (Barske et al., 2016). The Jagged-Notch signal is also involved in patterning the aggregation position of osteochondral progenitors, along with the Fgf signal (Paudel et al., 2022).

In all examined vertebrates, Sox9 is a master regulator of chondrogenic differentiation. Consistently, loss-of-function mutations in *sox9a* cause severe defects in facial cartilages, including HM, in zebrafish (Yan et al., 2002). Sox9 expression is regulated by integrated multiple signaling molecules, including positive Hedgehog, PTHrP, Bmp, Tgfβ, Fgf, and non-canonical Wnt and inhibitory canonical Wnt, Notch, and retinoic acid (Kozhemyakina et al., 2015). Recently, *barx1*-expressing mesenchyme that co-expresses *sox9a* in the anterior region of HM plate at 48 hpf has been described in zebrafish, though a genetic requirement for this has yet to be identified (Paudel et al., 2022).

Although key regulators of facial cartilage development have been identified, the specific genetic mechanisms involved in HM formation remain unexplored in fish. In zebrafish, defects in the first pouch that segments the mandibular and hyoid arches have been associated with specific defects in HM cartilage, as evidenced by a mutation in *integrin-α-5* (*itg-α-5*) that has shown particular defects in the first pouch and HM cartilage (Crump et al., 2004b). Similarly, the loss of *tbx1* or *fgf8a*, as well as the simultaneous loss of two EphrinB ligands (*efnb2a* and *efnb3b*) or two Pax1 paralogs (*pax1a* and *pax1b*), have led to abnormal development of the pouches and facial cartilage, including related defects in the first pouch and HM cartilage (Piotrowski and Nüsslein-Volhard, 2000; Piotrowski et al., 2003; Crump et al., 2004a; Choe and Crump, 2015; Liu et al., 2020). However, how the first pouch directs HM cartilage development is still unclear.

During the evolution of protochordate, the endodermal expression of the *pax1/9* gene has been implicated in craniofacial development, such as gill formation (Ogasawara et al., 2000; Liu et al., 2015). In vertebrates, the *pax1/9* gene underwent duplication, evolving into *pax1* and *pax9* (Balczarek et al., 1997), with endodermal expressions *pax1* and *pax9* essential for craniofacial development, including facial skeletal development, in fish and mice, respectively (Peters et al., 1998; Okada et al., 2016; Liu et al., 2020). In medaka, *pax1* is expressed in the pharyngeal endoderm and is essential for developing pouches and related facial cartilages (Okada et al., 2016). In zebrafish, two paralogs of *pax1* are co-expressed in the pharyngeal endoderm and act redundantly to develop the pouches, as well as the facial cartilages derived from the hyoid and gill arches (Liu et al., 2020). Genetically, Pax1 acts upstream of Tbx1 and Fgf3 in pouch formation in both medaka and zebrafish (Okada et al., 2016; Liu et al., 2020). In mice, *pax9* is expressed in the pharyngeal endoderm and is required to develop the posterior pouches and facial skeletal elements (Peters et al., 1998). However, in fish and mice, defects in facial skeletal elements by loss of *pax1* or *pax9* are primarily considered to be secondary effects due to pouch defects. Here, we report a direct role for *pax1a*, expressed in the first pouch, in regulating HM cartilage development by promoting the survival of osteochondral progenitors in the adjacent hyoid arch through the EfnB2a signal.

## Materials and methods

### Zebrafish lines

All zebrafish (*Danio rerio*) were handled as described previously (Kimmel et al., 1995) and approved by the Gyeongsang National University Institutional Animal Care and Use Committee. Published lines include *Tg(her5:mCherryCAAX)* ^*el72*^ (Choe et al., 2013) and *Tg(sox10:EGFP)* ^*ba2*^ (Carney et al., 2006). *Tg(sox17:EGFP)*, *Tg(sox17:Gal4VP16)*, *Tg(UAS:Pax1a)*, *Tg(UAS:Efnb2a)*, *Tg(UAS:EGFP)*, *Tg(hsp70I:Pax1a)* and *Tg(hsp70I:Efnb2a)* transgenic constructs were generated using the Gateway (Invitrogen) Tol2kit (Kwan et al., 2007). For p5E-*sox17*, PCR amplicons covering approximately 5 kb of upstream regulatory regions were acquired with primers *sox17*-B4F and *sox17*-B1R from multi-stage zebrafish genomic DNA. For pME-Pax1a, the coding sequence of *pax1a* was amplified using primers Pax1a-B1F and Pax1a-B2R from multi-stage zebrafish cDNA. pME-Efnb2a was published (Choe and Crump, 2015). Three independent transgenic lines for *Tg(sox17:Gal4VP16:pA)* were secured based on *cmlc2*:GFP heart fluorescence. Two independent transgenic lines for *Tg(UAS:Pax1a:pA)*, two for *Tg(UAS:Efnb2a:pA)*, three for *Tg(hsp70I:Pax1a:pA)*, and two for *Tg(hsp70I:Efnb2a:pA)* were established based on *α-crystallin*:Cerulean eye fluorescence. *Tg(sox17:Gal4VP16:pA)* ^*GNU99*^, *Tg(UAS:Pax1a:pA)* ^*GNU102*^, *Tg(UAS:Efnb2a:pA)* ^*GNU100*^, *Tg(hsp70I:Pax1a:pA)* ^*GNU111*^, and *Tg(hsp70I:Efnb2a:pA)* ^*GNU112*^ were used for this study.

Mutant lines for *pax1a, pax1b*, and *efnb2a* were generated with CRISPR/Cas9 system in wild-type Tübingen embryos. One mutant allele for *pax1a* (*pax1a* ^*GNU25*^), one allele for *pax1b* (*pax1b* ^*GNU28*^), and two alleles for *efnb2a* (*efnb2a* ^*GNU89*^ and *efnb2a* ^*GNU90*^) were established. For genotyping of *pax1a* ^*GNU25*^, PCR amplicons produced by primers *pax1a*-GT_F and *pax1a*-GT_R were treated with BsrI; while the wild-type allele produced 362 bp, the *pax1a* mutant allele generated 112 and 240 bp. For genotyping of *pax1b* ^*GNU28*^, PCR fragments obtained using primers *pax1b*-GT_F and *pax1b*-GT_R were digested with MspA1I; the wild-type allele generated 264 bp, whereas *the pax1b* mutant allele produced 117 and 142 bp. Genotyping of *efnb2a* ^*GNU89*^ and *efnb2a* ^*GNU90*^ was performed using PCR with primers *efnb2a*-GT_F and *efnb2a*-GT_R, followed by digestion with BsrBI and MspI, respectively. The wild-type allele remains uncut, whereas the *efnb2a* ^*GNU89*^ allele is cleaved into 64 bp and 282 bp fragments, and the *efnb2a* ^*GNU90*^ allele into 62 bp and 275 bp fragments. See Supplementary Materials and Methods for primers.

### Quantitative real-time PCR

Total RNA was extracted from the wild type and each gene mutant at 48 hpf using the NucleoSpin RNA Plus kit (MACHEREY-NAGEL). Approximately 30–40 embryos were used for each RNA extraction. RNA quality and quantity were assessed using a NanoDrop spectrophotometer to ensure A260/A280 ratios between 1.8 and 2.0. cDNA was synthesized with 1 µg of total RNA using ReverTra Ace™ High Efficient Reverse Transcriptase (TOYOBO). Quantitative real-time PCR (qRT-PCR) was conducted using the SsoFast EvaGreen Supermix (Bio-Rad) on a Rotor-Gene Q (QIAGEN) PCR machine with the following cycling conditions: an initial denaturation at 95°C for 5 min, followed by 40 cycles of 95°C for 10 s and 60°C for 30 s. Each reaction was performed in triplicate to ensure technical accuracy. Ct values and relative gene expression were calculated using the ^**ΔΔ**^ **Ct Relative Quantification method** (QIAGEN). *β-actin* was used as an internal control to normalize the expression levels, with *β-actin* real-time primer used previously (Jeon et al., 2022). Statistical analysis was performed using GraphPad Prism. Data were analyzed using an unpaired t-test, with significance determined at *p* < 0.05.

### Heat shock of larval zebrafish

Heat shock was performed as described for 40 min (Scott et al., 2017). After the heat shock, tubes were removed from the water bath and placed into a 28.5°C incubator to cool gradually. Following recovery, embryos were fixed in 4% paraformaldehyde for subsequent analysis. No developmental or morphological defects were observed in heat-shocked embryos.

### Staining

Alcian blue and alizarin red staining, GFP immunohistochemistry (Torrey Pines Biolabs, 1:1000), fluorescent double *in situ* hybridizations, and Lysotracker and BrdU staining were carried out as described (Zuniga et al., 2011; Choe and Crump, 2014). TUNEL assay was performed according to the protocol described by Cole and Ross (2001). Riboprobes to *tbx1*, *fgf3*, *itg-α-5*, *efnb2a,* and *efnb3b* were published (Crump et al., 2004b; Choe and Crump, 2014; 2015). For riboprobes to *pax1a*, *pax1b*, *barx1*, and *sox9a*, PCR amplicons were cloned into pGEM^®^-T Easy Vector Systems (Promega), linearized, and then digoxigenin- or dinitrophenol-labeled RNAs were synthesized using T7 or SP6 RNA polymerase (Roche). See Supplementary Materials and Methods for primers.

### Imaging

Craniofacial cartilages were dissected manually with fine insect pins, flat-mounted, and photographed on an Olympus BX50 upright microscope using mosaic V2.1 software. Fluorescent images were taken on an Olympus FV3000 confocal microscope using FV31S-SW software. After capturing approximately 80 μm Z-stacks at 3.0 and 1.5 μm intervals with Olympus UPLXAPO ×20 and ×40 objective lenses, respectively, maximum intensity projections encompassing static confocal sections were assembled using FV31S-SW software. Any adjustments were applied to all panels using Adobe Photoshop.

### Statistics

The numbers of HM cells and BrdU-, Lysotracker Red-, and TUNEL Red-positive cells were manually counted to quantify HM defects and cell proliferation and death. Three independent individuals cross-verified the counting of HM cells. We employed the multiple comparison tests of Tukey–Kramer to quantify HM defects and cell proliferation and death and a chi-square test to compare the frequency of groups quantitatively.

## Results

### The expression of *pax1a* and *pax1b* continues in the mature pouches after arch segmentation stage

**FIGURE 1:**
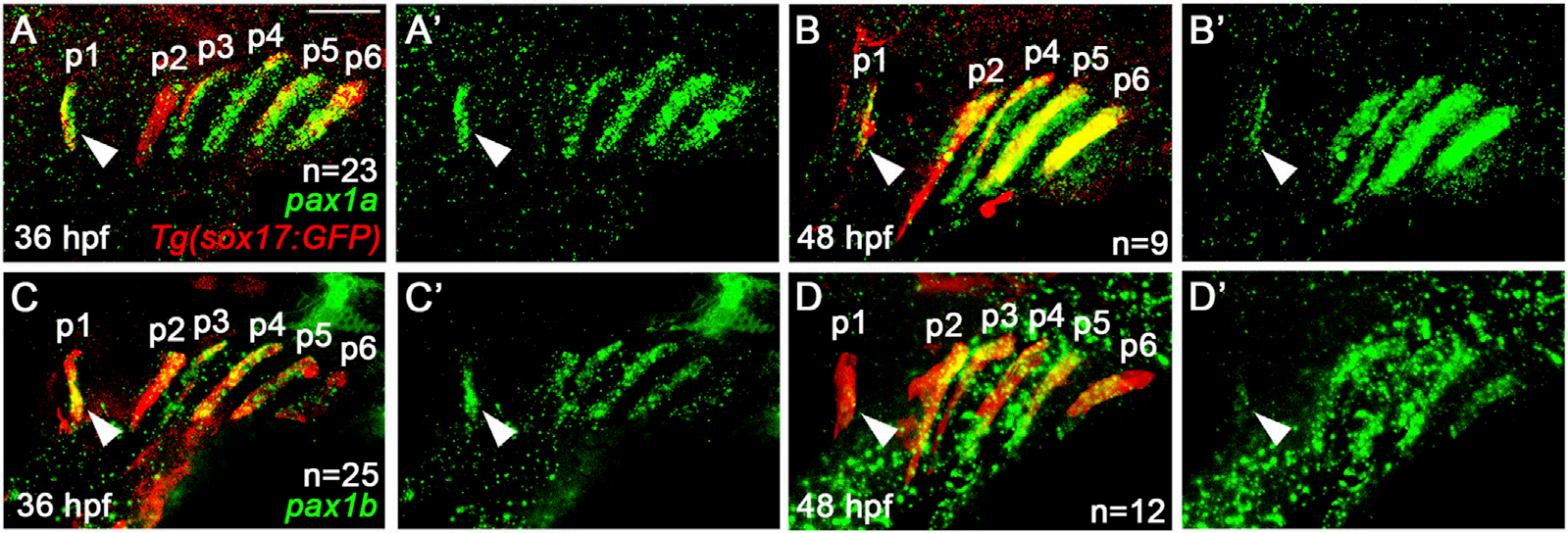
Expression of *pax1a* and *pax1b* in the mature pouches. **(A–D)** Fluorescence *in situ* hybridization of *pax1a* and *pax1b* (green) in conjunction with the GFP immunohistochemistry (red) in wild-type *Tg(sox17:GFP)* reporter lines marking the pharyngeal endoderm and pouches. Pouches are marked as p1-p6. Arrowheads indicate the expression of *pax1a* and *pax1b* in the first pouch (p1). **(A, C)** At 36 hpf, transcripts for *pax1a* and *pax1b* are seen in *sox17*-positive pouches. **(B, D)** At 48 hpf, the expression of *pax1a* and *pax1b* continues in *sox17*-positive pouches. **(A′–D′)** Green channel only. Scale bar: 40 μm. Anterior is to the left.

Previously, it was reported that *pax1a* and *pax1b* are expressed in the pharyngeal endoderm at the stage of arch segmentation, with their redundant role in the morphogenesis of pharyngeal pouches (Liu et al., 2020). Although it was shown that *pax1a* and *pax1b* expression continues in the embryonic head by 96 hpf, their expression domain has yet to be clearly determined (Liu et al., 2020). To get an insight into a late role for Pax1 in facial skeletal development, we examined the expression of *pax1a* and *pax1b* at 36 and 48 hpf. When arch segmentation was completed by the pouches at 36 hpf, *pax1a* and *pax1b* were still expressed in the mature pouches, with their expression continued at 48 hpf (Figure 1). Considering 36 and 48 hpf are critical time points for the cranial neural crest-derived mesenchyme in the arches to differentiate into the chondrocytes to form facial cartilages (Barske et al., 2016), the late expression of *pax1a* and *pax1b* in the mature pouches suggests a distinct role of Pax1 in facial skeletal development that differs from its early role in pouch formation.

### Generation of loss-of-function mutations in *pax1a* and *pax1b* genes

To access a role for *pax1a* and *pax1b* in the development of facial cartilages, we generated loss-of-function mutations in the *pax1a* and *pax1b* genes with the CRISPR/Cas9 system. For *pax1a* mutants, we established a ten-nucleotide deletion allele in the *pax1a* gene (*pax1a* ^*GNU25*^) compared to the wild-type allele (Supplementary Figure S1A). While the wild-type allele produces 359 amino acids, the mutant allele was predicted to produce only 15 normal amino acids and an extra 11 amino acids, with most of the paired box domain (PBD) missing due to a premature stop codon (Supplementary Figure S1A). Moreover, the mRNA levels of *pax1a* were significantly reduced in *pax1a* mutants compared to the wild type (Supplementary Figure S1C). Thus, *pax1a* ^*GNU25*^ is a loss-of-function allele. For *pax1b* mutants, we secured a five-nucleotide deletion allele in the *pax1b* gene (*pax1b* ^*GNU28*^) (Supplementary Figure S1B). Compared to the wild-type *pax1b* allele, producing 340 amino acids, the *pax1b* ^*GNU28*^ allele was predicted to make 17 normal amino acids and an extra 57 amino acids, with most PBD missing (Supplementary Figure S1B). The mRNA levels of *pax1b* were also reduced by about 50% in *pax1b* mutants compared to the wild type (Supplementary Figure S1C). Thus, we suggest that *pax1b* ^*GNU28*^ is a loss-of-function allele.

### Pax1a plays a distinct role from Pax1b in hyomandibular cartilage development

**FIGURE 2:**
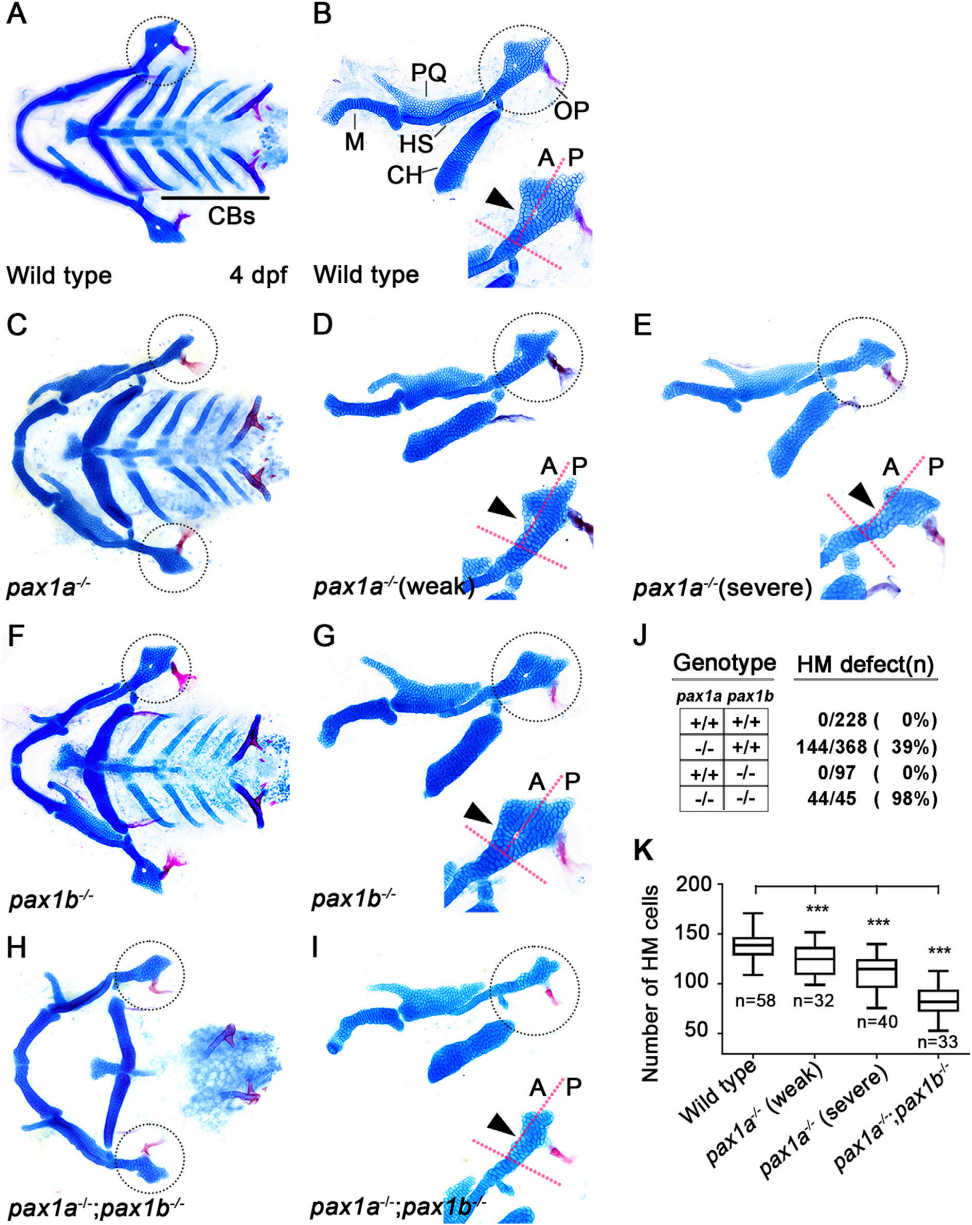
Requirement of *pax1a* in hyomandibular cartilage development. **(A–I)** Craniofacial skeletal elements stained with Alcian Blue (cartilage) and Alizarin Red (bone) at 4 dpf, with the hyomandibular (HM) plate circled. **(A, C, F, H)** Ventral views of dissected facial cartilages. **(B, D, E, G, I)** Lateral views of dissected facial skeletal elements of the mandibular and hyoid arches, along with inserts showing the anterior and posterior regions of HM (circled areas). **(A, B)** All facial skeletal elements are present in wild types, including the triangular HM plate, with an arrowhead indicating the multi-layered cells in the anterior region of HM. Five CBs on one side are underlined. **(C–E)** In *pax1a* mutants, a specific defect is seen in the HM plate, with other skeletal elements unaffected. Arrowheads indicate mono-layered cells **(D)** and missing cells **(E)** in the anterior region of HM. **(F, G)** In *pax1b* mutants, all facial skeletal elements are normal, including the HM plate. The normal multi-layered cells in the anterior region of HM are marked with an arrowhead **(H, I)** In double mutants for *pax1a* and *pax1b*, the HM plate is defective, and most CB cartilages are missing. Arrowhead indicates the missing cells in the anterior region of HM. M, Meckel’s cartilage; PQ, Palatoquadrate cartilage; HS, Hyosymplectic cartilage; CH, Ceratohyal cartilage; CB, Ceratobranchial cartilage; OP, Opercle bone; A, anterior; P, posterior. Anterior is to the left. **(J)** The number of mutants analyzed and the penetrance for HM defects. **(K)** Quantification of the number of cells in HM. Data is represented on a boxplot. *** shows *p* < 0.001.

In zebrafish, single mutants for *pax1a* or *pax1b* did not affect the development of facial cartilages, whereas double mutants for *pax1a* and *pax1b* showed severe defects in the HM plate and ceratobranchial (CB) cartilages whose development relied on the pouches (Liu et al., 2020). However, in *pax1a* mutants, we observed defects in the HM plate, but with other facial cartilages, including CBs, being normal (Figures 2C–E). In wild-type animals, the HM plate is shaped like a triangle bearing a foramen in which the fifth facial nerve passes (Iwasaki et al., 2022; Figures 2A, B). The HM plate can be divided into anterior and posterior parts based on the foramen position (Iwasaki et al., 2022), with multi-layered cells composing the anterior part adjacent to the foramen (arrowhead in Figure 2B). In *pax1a* mutants, the HM plate bore the foramen, but only mono-layered cells formed the anterior part adjacent to the foramen (arrowhead in Figure 2D). These defects were classified as weak phenotypes. Eventually, the foramen and the anterior part were missing from the deformed HM plate in *pax1a* mutants (arrowhead in Figure 2E), and these defects were classified as severe phenotypes. Consistently, the number of cells in the HM plates was significantly decreased in *pax1a* mutants compared to wild-type animals (Figure 2K; Supplementary Table S1).

Since it has been shown that in/del mutation generated by the CRISPR/Cas9 system can lead to phenotypes that are weaker than either point mutation or morpholino-mediated knockdown due to a genetic compensation (Rossi et al., 2015; El-Brolosy et al., 2019), we examined *pax1b* expression in *pax1a* mutants with qRT-PCR. Indeed, we observed that the mRNA levels of *pax1b* increased by about 1.5 folds in *pax1a* mutants (Supplementary Figure S1D). Nonetheless, the HM defects in *pax1a* mutants suggest a distinct role of Pax1a in HM development, which Pax1b cannot entirely compensate. In contrast, as previously reported, any defects in facial cartilages, including the HM plate and CBs, were not seen in *pax1b* mutants (Figures 2F, G), in which the mRNA levels of *pax1a* increased in about 12 folds (Supplementary Figure S1E). This result implies that the Pax1b function can be replaced with Pax1a in the development of HM plate and CB cartilages.

Confirming a high degree of genetic redundancy of Pax1a and Pax1b in facial cartilage formation, the HM was distorted as a rod, with most CBs missing, in double mutants for *pax1a* and *pax1b* (Figures 2H, I), in which the mRNA levels of *pax1a* and *pax1b* were significantly reduced (Supplementary Figure S1C). In particular, in the double mutants, HM defects were more severe, with the number of cells in the HM plates significantly decreasing, and the genetic penetrance for the phenotype increased compared to *pax1a* mutants (Figures 2J, K). Despite a degree of genetic redundancy of Pax1a and Pax1b in the development of the HM plate, our data indicate a distinct role of Pax1a in the development of the HM plate that cannot be replaced with Pax1b.

### Pax1a partially controls the development of the HM plate through the formation of the first pouch

Previously, it was reported that the first pouch is essential for HM plate development in the hyoid arch (Crump et al., 2004b), with Pax1a and Pax1b acting redundantly to form pouches, including the first pouch (Liu et al., 2020). Since we observed defects in the HM plate in *pax1a* mutants, we examined the first pouch in the mutants. To do so, we analyzed the first pouch and HM plate simultaneously in the same individual, using wild-type and mutant animals bearing *Tg(her5:mCherryCAAX)* and *Tg(sox10:GFP)* transgenes that visualize the pouches and arches in real-time. *pax1a* mutant individuals with first pouch defects at 34 hpf displayed defects in the HM plate at 4 dpf (Supplementary Figure S2). This result indicates that Pax1a is required to develop the first pouch to control the development of the HM plate.

Next, we addressed whether Pax1a-dependent first pouch formation is sufficient for the HM plate development. To do so, we tried to rescue the defects in the HM plate by rescuing the first pouch formation in double mutants for *pax1a* and *pax1b*, which shows almost complete penetrance of HM defects (Figure 2J). Since the first pouch formation begins at 16 hpf and is completed at 18 hpf (Choe et al., 2013), we expressed the wild-type *pax1a* gene at 16 hpf for 40 min with a heat-shock approach in double mutants for *pax1a* and *pax1b*. At 17 hpf, *pax1a* expression was nearly absent in double mutants for *pax1a* and *pax1b* compared to wild types, and it was not induced by the heat-shock treatment (Supplementary Figures S3A–C). However, in double mutants for *pax1a* and *pax1b* carrying *Tg(hsp70I:Pax1a)* transgene, *pax1a* was overexpressed in all areas of the embryos, including the pharyngeal regions, by the heat-shock treatment (Supplementary Figure S3D).

**FIGURE 3:**
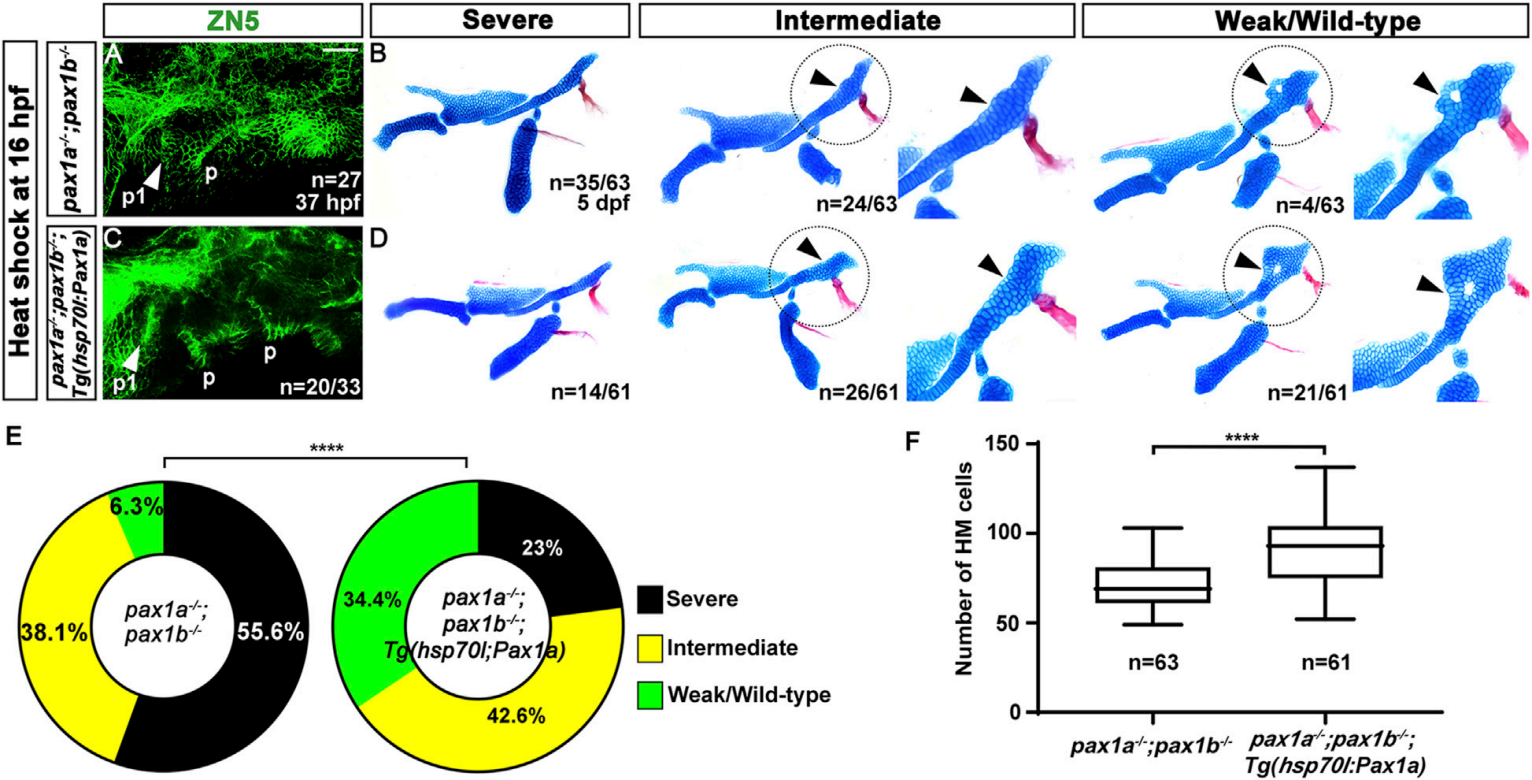
Rescue of the defects in the first pouch and hyomandibular cartilage in *pax1* double mutants with an enforced expression of *pax1a* at 16 hpf. **(A, C)** Zn5 staining labels to visualize pouches at 37 hpf. The first pouches are marked with arrowheads. After the heat-shock treatment at 16 hpf for 40 min, while only two pouches (p1 and p) with abnormal shapes are seen in *pax1* double mutants **(A)**, the first pouch (p1) is pretty normal, with other pouches disorganized, in *pax1* double mutants carrying *Tg(hsp70I:Pax1a)* transgene **(C)**. p, pouch. Scale bar = 40 μM. **(B, D)** Unilateral dissections of the skeletons of the mandibular and hyoid arches stained with Alcian Blue (cartilage) and Alizarin Red (bone) at 5 dpf, along with inserts showing the zoomed-in HM plate (circled areas). **(B)** After the heat shock treatment in *pax1* double mutants, a rod-shape HM is seen in the severe group, more cells appear in the rod-shape HM in the intermediate group compared to the severe group, and the foramen is seen, with adjacent mono-layered cells in the weak group. Arrowheads indicate the cells appearing in the HM plate in the intermediate group and the mono-layered cells adjacent to the foramen in the weak group. No wild-type HM plate is observed. **(D)** After the heat shock treatment in *pax1* double mutants bearing *Tg(hsp70I:Pax1a)* transgene, HM defects in all three groups were observed, but with a different frequency than those observed in **(B)**; wild-type HM plates are also observed. Arrowheads indicate the cells appearing in the HM plate in the intermediate group and the multi-layered cells adjacent to the foramen in the wild-type HM plate. **(E)** Quantification of the frequency of each group between **(B, D)**. The frequency of each group is counted. Data is represented on a pie chart. Black, yellow, and green represent the severe, intermediate, and weak/wild-type groups, respectively. **** indicates *p* < 0.0001. **(F)** Quantification of HM defects. The number of cells in HM is counted. Data is represented on a boxplot. **** indicates *p* < 0.0001. n, number of animals analyzed.

After the heat-shock treatment at 16 hpf for 40 min, all double mutant siblings carrying no *Tg(hsp70I:Pax1a)* transgene displayed defects in the first pouch at 37 hpf (arrowhead in Figure 3A), as shown by ZN5 staining, a molecular marker for pharyngeal pouches (Piotrowski and Nüsslein-Volhard, 2000). However, about 60.6% (n = 20/33) of double mutant siblings carrying *Tg(hsp70I:Pax1a)* transgene showed normal development of the first pouch at 37 hpf (arrowhead in Figure 3C), verifying the role of Pax1a in the first pouch formation. After the heat-shock treatment, about 6.3% (n = 4/63) of double mutant siblings bearing no *Tg(hsp70I:Pax1a)* transgene showed weak defects in the HM plate in which the foramen was seen with mono-layered cells composing the anterior part adjacent to the foramen. However, about 93.6% (n = 59/63) of animals showed intermediate to severe defects in the HM plate, which was characterized by the absence of the foramen and anterior part, resulting in a plate-like or rod-shaped HM, respectively (Figure 3B). In contrast, after the heat-shock treatment, about 34.4% (n = 21/61) of double mutant siblings bearing *Tg(hsp70I:Pax1a)* transgene showed wild-type (n = 11/21) or weak defects in the HM plate (n = 10/21), and about 65.6% (n = 40/61) animals showed intermediate or severe defects in the HM plate (Figure 3D). The increase in the degree of the weakly defective or wild-type HM plate in the double mutants by the heat shock was statistically significant and was due to the increase in the number of cells composing the HM plate (Figures 3E, F; Supplementary Table S1). This result suggests a rescue of the first pouches and HM plate in the double mutants by inducing *pax1a* expression at 16 hpf, which confirms the critical role of Pax1a in the HM development through the first pouch formation. Interestingly, however, we observed that in the double mutants carrying *Tg(hsp70I:Pax1a)* transgene treated by the heat shock, about 60.6% (n = 20/33) of animals showed rescued first pouch at 37 hpf, but only about 34.4% (n = 21/61) of animals showed rescued HM plate at 5 dpf. This discrepancy in rescuing the first pouch and HM plate may suggest that, in the HM plate development, Pax1a-dependent first pouch formation is essential, but more is needed.

### Pax1a can regulate HM plate development independently of the first pouch formation

The late expression of *pax1a* in the pouches at 36 and 48 hpf and the discrepancy in rescuing the first pouch and HM plate suggest that Pax1a has a distinct role in HM plate development independent of the first pouch formation. To test this hypothesis, we addressed whether the late expression of *pax1a* at 36 hpf after pouch formation can rescue the defects in the HM plate in the double mutants for *pax1a* and *pax1b*. Compared to wild types, in double mutants for *pax1a* and *pax1b* at 37 hpf, *pax1a* expression was significantly reduced, and its expression was not induced by the heat-shock treatment at 36 hpf for 40 min (Supplementary Figures S3E–G). After the heat-shock treatment at 36 hpf for 40 min, *pax1a* was overexpressed in most areas of the embryos, including the pharyngeal regions in double mutants for *pax1a* and *pax1b* carrying *Tg(hsp70I:Pax1a)* transgene (Supplementary Figure S3H).

**FIGURE 4:**
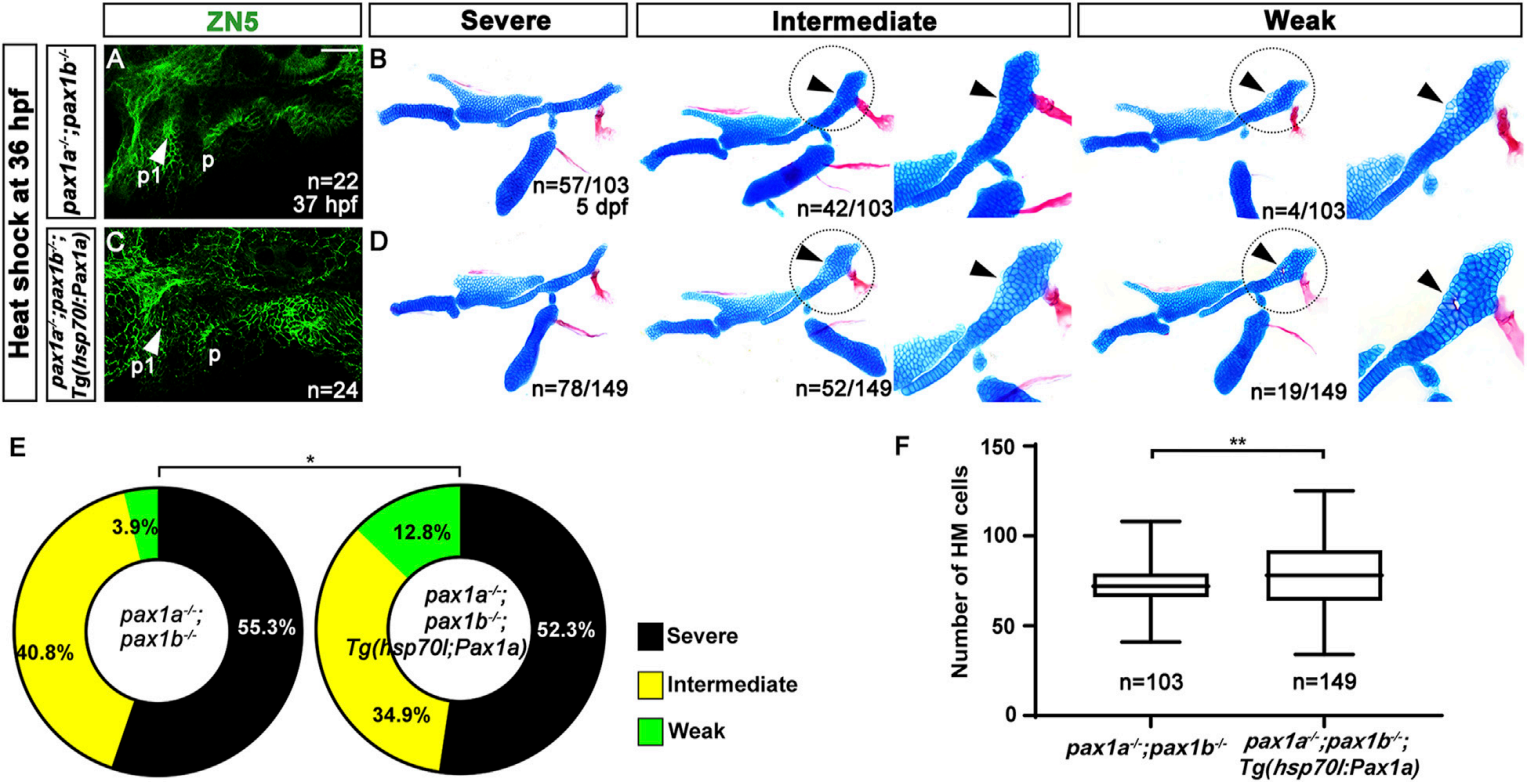
Rescue of the defects in the hyomandibular cartilage in *pax1* double mutants with an enforced expression of *pax1a* at 36 hpf. **(A, C)** Zn5 immunohistochemistry to visualize pouches at 37 hpf. Arrowheads mark pouches. After the heat-shock treatment at 36 hpf for 40 min, *pax1* double mutants display two pouches (p1 and p) with abnormal shapes **(A)**, and *pax1* double mutants carrying *Tg(hsp70I:Pax1a)* transgene show a distorted pouch (p), with the first pouch (p1) barely seen **(C)**. Scale bar = 40 μM. **(B, D)** Lateral views of dissected facial skeletal elements of the mandibular and hyoid arches, along with inserts showing the zoomed-in HM plate (circled areas). After the heat shock treatment, the severe, intermediate, and weak defects in HM are seen in both *pax1* double mutants and those bearing *Tg(hsp70I:Pax1a)* transgene. No wild-type HM plate is observed in **(B, D)**. Cells appearing in the HM plate in the intermediate group and the mono-layered cells adjacent to the foramen in the weak group are indicated by arrowheads. **(E)** Quantification of the frequency of each group between **(B, D)**. The frequency of each group is counted. Data is represented on a pie chart. Black, yellow, and green represent the severe, intermediate, and weak groups, respectively. * indicates *p* < 0.05. **(F)** Quantification of HM defects. The number of cells in HM is counted. Data is represented on a boxplot. ** indicates *p* < 0.01. n, number of animals analyzed.

In *pax1a* and *pax1b* double mutants bearing no transgene and their siblings carrying *Tg(hsp70I:Pax1a)* transgene, all analyzed animals showed the first pouch was malformed or barely seen after the heat-shock treatment, indicating the induction of *pax1a* expression at 36 hpf in the double mutants could not rescue the first pouch formation (Figures 4A, C). After the heat-shock treatment, about 3.9% (n = 4/103) of double mutant siblings bearing no *Tg(hsp70I:Pax1a)* transgene displayed weak defects in the HM plate in which the foramen was seen with mono-layered cells composing the anterior part adjacent to the foramen, with about 96.1% (n = 99/103) animals showing intermediate or severe defects in the HM plate in which the foramen and anterior part were missing (Figure 4B). Interestingly, however, after the heat-shock treatment, about 12.8% (n = 19/149) of double mutant siblings bearing *Tg(hsp70I:Pax1a)* transgene showed weak defects in the HM plate, and about 87.2% (n = 130/149) animals showed intermediate or severe defects in the HM plate (Figure 4D). We did not observe the complete wild-type HM plate by the heat-shock treatment in both *pax1a* and *pax1b* double mutants carrying *Tg(hsp70I:Pax1a)* transgene and their siblings bearing no transgene. Although the increase in the degree of the weakly defective HM plate in the double mutants by the heat shock at 36 hpf was not as dramatic as that by the heat shock at 16 hpf, it was still statistically significant (Figure 4E). In addition, after the heat shock, the number of cells composing the HM plate increased in the double mutants carrying *Tg(hsp70I:Pax1a)* transgene compared to the double mutant siblings carrying no transgene (Figure 4F; Supplementary Table S1). This result suggests a partial rescue of the HM plate in the double mutants by inducing *pax1a* expression at 36 hpf, indicating a distinct role of Pax1a in the HM development, independent of the first pouch formation.

### Pax1a is necessary for the survival of osteochondral progenitors differentiating into the chondrocytes in the hyoid arch

**FIGURE 5:**
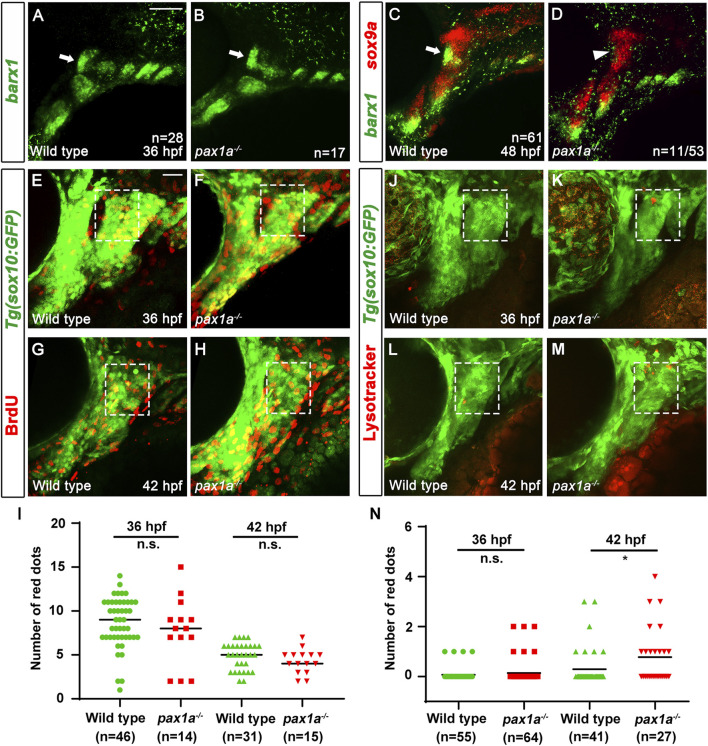
Loss of skeletogenic mesenchyme in the anterior region of developing hyomandibular plate in *pax1a* mutants. **(A, B)** Fluorescent *in situ* hybridization for *barx1* (green) at 36 hpf shows almost identical condensations of osteochondral progenitors in the dorsal region of the hyoid arch of wild types and *pax1a* mutants (arrows). **(C, D)** Double *in situ* hybridization for *barx1* (green) and *sox9a* (red) at 48 hpf. **(C)** In wild types, chondrocytes partially co-expressing *barx1* and *sox9a* and *barx1*-expressing mesenchymal cells are observed in the anterior region of the developing HM (arrow). **(D)** In 11 of 53 *pax1a* mutants, those chondrocytes and mesenchymal cells are not seen in the anterior region of the developing HM (arrowhead). Scale bar = 40 μM. Anterior is to the left. Dorsal is at the top. **(E–H)** BrdU staining (red) visualizes proliferating cells relative to *Tg(sox10:GFP)* expressing mesenchymal cells (green) in wild-type siblings and *pax1a* mutants. The dorsal regions of the hyoid arches are boxed. The upper and lower boundaries of the box are set along the dorsal and ventral ends of the first pouch. The anterior and posterior boundaries are defined between the first pouch anteriorly and the core mesoderm posteriorly located at the center of the hyoid arch. **(I)** Quantification of the number of proliferating cells in the dorsal region of the hyoid arch [boxed in **(E–H)**]. Data is represented on a scatter plot. n.s., not significant. **(J–M)** Lysotracker Red staining (red) labels dying cells relative to *Tg(sox10:GFP)* expressing mesenchymal cells (green) in wild-type siblings and *pax1a* mutants. The dorsal regions of the hyoid arches are boxed, which are set with the same definition as in **(E–H)**. Scale bar = 20 μM. Anterior is to the left. Dorsal is at the top. **(N)** Quantification of the number of dying cells in the dorsal region of the hyoid arch [boxed in **(E–H)**]. Data is represented on a scatter plot. * shows *p* < 0.05. n.s., not significant. n, number of animals analyzed.

The decrease in the number of cells in the HM plate of *pax1a* mutants, with the increase in cell numbers by inducing *pax1a* expression at 36 hpf in the double mutants carrying *Tg(hsp70I:Pax1a)* transgene suggests the role of Pax1a in the HM plate development would be involved in the survival or proliferation of cells contributing to the HM cartilages. To understand the cellular requirements of Pax1a at 36 and 48 hpf in the development of the HM plate, we analyzed the developmental processes of HM cartilage with molecular markers in *pax1a* mutants. In wild types, *barx1* expression at 36 hpf marks condensed osteochondral progenitors in the dorsal region of the hyoid arch, which will further differentiate into the chondrocytes contributing to the HM plate (Barske et al., 2016). Compared to wild types, *barx1* expression at 36 hpf in the dorsal regions of the hyoid arch was unaffected in *pax1a* mutants, indicating that Pax1a is not essential for the condensation of osteochondral progenitors in HM plate formation (arrows in Figures 5A, B). In wild types, *barx1* expression continues in mesenchymal cells that are partially overlapped with *sox9a*-expressing chondrocytes in the dorsal region of the hyoid arch at 48 hpf (Paudel et al., 2022; arrow in Figure 5C). In *pax1a* mutants, while *sox9a* expression marking chondrocytes at 48 hpf was fairly normal in the dorsal region of the hyoid arch, *barx1* expression marking mesenchyme was almost abolished, with chondrocytes co-expressing *barx1* and *sox9a* not seen anymore (arrowhead in Figure 5D). This result suggests that Pax1a is required to form *barx1*-positive mesenchyme and *barx1*-and *sox9a*-co-expressing chondrocytes at 48 hpf.

Next, we analyzed the cellular responses of mesenchymal cells in the dorsal region of the hyoid arch during the specification of chondrocytes. Since it was reported that cell proliferation is barely seen in the hyoid arch at 48 hpf during facial skeletal development (Barske et al., 2016; Paudel et al., 2022), we examined cell proliferation in the hyoid arch at 36 and 42 hpf with BrdU staining. In wild types and *pax1a* mutants, we observed a similar level of proliferating cells in the hyoid arch at 36 and 42 hpf (Figures 5E–I; Supplementary Table S1). Then, we focused on analyzing cell survival in the hyoid arch in *pax1a* mutants with Lysotracker and TUNEL staining. In wild types, cell death was barely observed in the condensations of osteochondral progenitors and during the specification of chondrocytes (boxed in Figures 5J, L; boxed in Supplementary Figures S4A, C; Supplementary Table S1). In *pax1a* mutants, little cell death was seen in the condensations (boxed in Figure 5K; boxed in Supplementary Figure S4B; Supplementary Table S1), but it increased during chondrocyte specification compared to wild types (boxed in Figures 5M, N; boxed in Supplementary Figures S4D, E; Supplementary Table S1). This result suggests that Pax1a is required to survive osteochondral progenitors during chondrocyte specification.

### EphrinB2a is genetically linked with Pax1a in HM plate development

Considering that Pax1a is a transcription factor, the limited expression of *pax1a* in the first pouch, not in the hyoid arch, indicates the presence of a mediator acting downstream of Pax1a to provide the survival signal to the HM progenitors in the hyoid arch. To identify the mediator, we first examined the expression of *tbx1*, *fgf3*, *itg-α-5*, *efnb2a,* and *efnb3b* that are expressed in the pouch endoderm for pouch formation (Crump et al., 2004b; Herzog et al., 2004; Choe and Crump, 2014; 2015). At 36 hpf, when the HM progenitors are condensing, *tbx1*, *fgf3*, and *itg-α-5* were still expressed in the posterior pouches but not in the first pouch (arrows in Supplementary Figures S5A–C), with *efnb3b* expression not seen in the pouches (Supplementary Figure S5F). The expression of *efnb2a* was observed in the pouches, with particularly strong expression in the first pouch at 36 hpf, and the expression patterns continued to 40 hpf (Supplementary Figures S5D, E).

**FIGURE 6:**
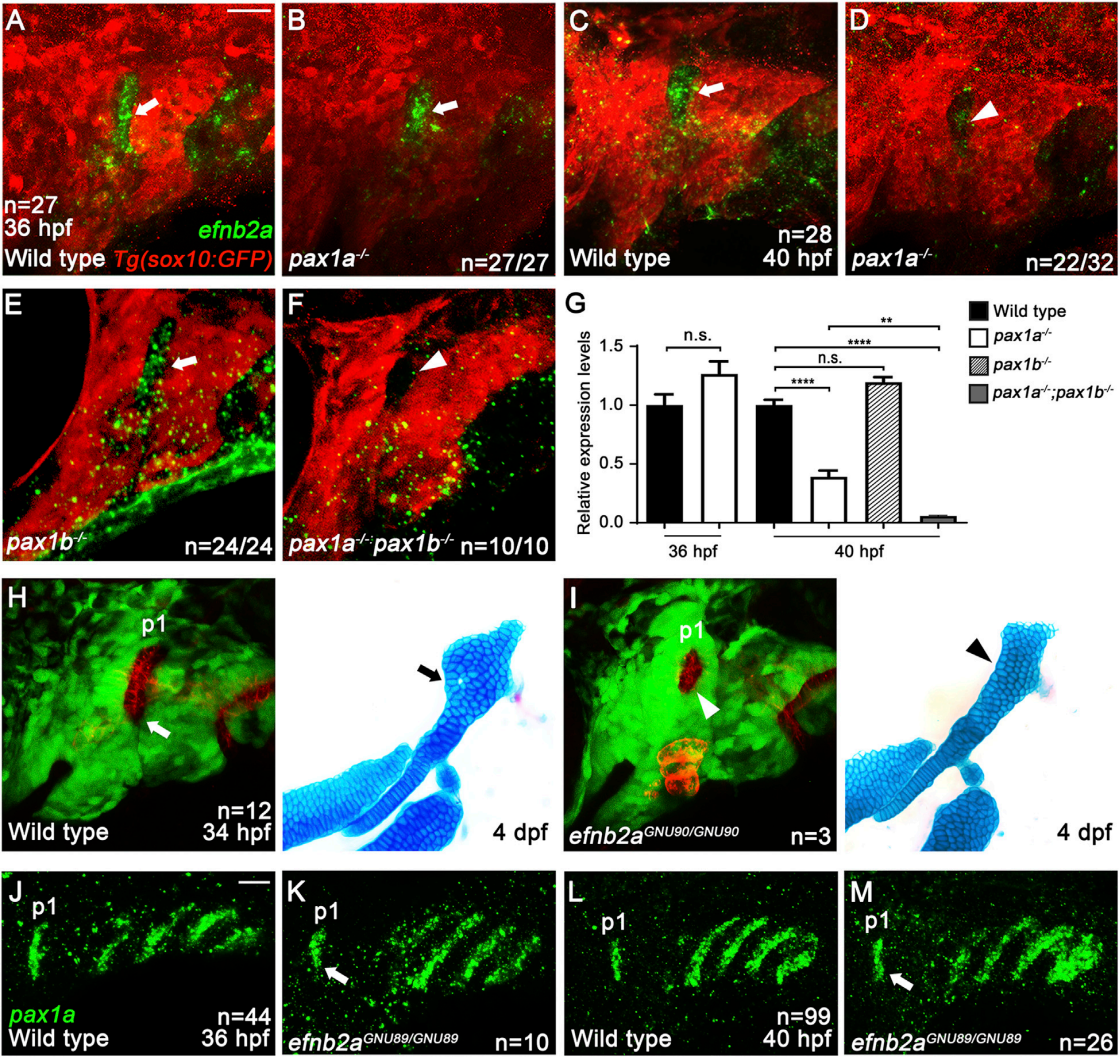
Genetic linkage of EphrinB2a with Pax1a in hyomandibular plate development. **(A–F)** *In situ* hybridization of *efnb2a* (green) in conjunction with the GFP immunohistochemistry (red). In wild types bearing *Tg(sox10:GFP)* transgene, *efnb2a* expression in the first pouch at 36 **(A)** and 40 hpf **(C)** is seen (arrows). In the first pouch of *pax1a* mutants, *efnb2a* expression at 36 hpf is unaffected [arrow in **(B)**] but is reduced at 40 hpf in 22 out of 32 animals [arrowhead in **(D)**]. In the first pouch of *pax1b* mutants, *efnb2a* expression at 40 hpf is unaffected [arrow in **(E)**]. In *pax1a* and *pax1b* double mutants, *efnb2a* expression in the first pouch at 40 hpf is abolished [arrowhead in **(F)**]. Scale bar = 20 μM. Anterior is to the left. **(G)** Relative expression levels of *efnb2a* mRNAs in wild types and *pax1* mutants. Expression in wild types set at 1. Transcripts of *efnb2a* are downregulated in *pax1a* mutants at 40 hpf but not at 36 hpf. Expression of *efnb2a* mRNAs is further reduced in *pax1a* and *pax1b* double mutants at 40 hpf compared to *pax1a* mutants. Transcripts of *efnb2a* are barely affected in *pax1b* mutants at 40 hpf compared to wild types. ** and **** show *p* < 0.01 and *p* < 0.0001, respectively. n.s., not significant. **(H, I)** Confocal projections from live imaging of embryos bearing *Tg(her5:mCherryCAAX)* (red) and *Tg(sox10:GFP)* (green) transgenes at 34 hpf, followed by Alcian Blue staining (blue) in the same individuals at 4 dpf. In a wild-type individual, a bilayered normal first pouch is seen at 34 hpf [white arrow in **(H)**], with the triangular HM plate observed at 4 dpf [black arrow in **(H)**]. In an *efnb2a* mutant individual, the first pouch was hypoplastic at 34 hpf [white arrowhead in **(I)**], with the HM plate defective at 4 dpf [black arrowhead in **(I)**]. **(J–M)** *In situ* hybridization for *pax1a* (green). Compared to wild types, *pax1a* expression in the first pouch was unaffected in *efnb2a* mutants at 36 and 40 hpf [arrows in **(K, M)**]. Scale bar = 20 μM. Anterior is to the left. n, number of animals analyzed.

Next, we analyzed whether the *efnb2a* expression in the first pouch at 36 and 40 hpf requires Pax1a. Compared to wild types (arrows in Figures 6A, C), *efnb2a* expression in the first pouch was unaffected at 36 hpf (arrow in Figure 6B), but it was downregulated at 40 hpf (arrowhead in Figure 6D), in *pax1a* mutants. The reduction in *efnb2a* expression observed in *pax1a* mutants with *in situ* hybridization was confirmed to be significant only at 40 hpf by qRT-PCR (Figure 6G). At 40 hpf, *efnb2a* expression in the first pouch was also significantly reduced in *pax1a* and *pax1b* double mutants (Figures 6F, G), whereas it was barely affected in *pax1b* mutants (Figures 6E, G). In particular, it was further reduced in the double mutants compared to *pax1a* mutants (Figure 6G). Considering the first pouch is adjacent to the dorsal region of the hyoid arch, this result implies that EfnB2a could act downstream of Pax1a in the first pouch to transmit a signal to adjacent skeletogenic mesenchymal cells in the dorsal region of the hyoid arch during HM plate formation. To test this hypothesis, we first determined the function of *efnb2a* in HM development. To do so, we established two loss-of-function alleles of the *efnb2a* gene (*efnb2a* ^*GNU89*^ and *efnb2a* ^*GNU90*^) with the CRISPR/Cas9 system (Supplementary Figures S5G, H). Although defects in the HM plate were not appreciated in *efnb2a* ^*hu3393*^ mutants in our previous study (Choe and Crump, 2015), we observed HM defects in *efnb2a* ^*GNU89*^ and *efnb2a* ^*GNU90*^ mutants with overall 12.8% (n = 6/47) genetic penetrance of the phenotype (Figures 6H, I; Supplementary Figures S5I, J). In part, the HM defects were associated with the malformed first pouches, as evidenced by the simultaneous analysis of pouches and facial cartilages in the same individual for *efnb2a* mutants (Figures 6H, I). Interestingly, the HM defects in *efnb2a* mutants phenocopied those in *pax1a* mutants in that the foramen and the anterior part adjacent are missing or defective. This result suggests a genetic linkage of Pax1a and EfnB2a in the development of the HM plate.

Indeed, double mutants for *pax1a* and *efnb2a* increased the severity of defects in the HM plate compared to single mutants for *pax1a* or *efnb2a* (Supplementary Figure S6). While 6.9% (n = 2/29) of the *pax1a* single mutant siblings and 2.6% (n = 1/38) of the *efnb2a* single mutant siblings showed severe defects in the HM plate, 44.5% (n = 8/18) of the double mutant siblings displayed severe defects in the HM plate (Supplementary Figure S6A). The increased severity observed in the double mutants was statistically significant, further supporting the genetic linkage of Pax1a and EfnB2a in the development of the HM plate (Supplementary Figure S6B). However, EfnB2a did not act upstream of Pax1a in the first pouch at 36 and 40 hpf, as *pax1a* expression in the first pouch was unaffected in *efnb2a* mutants (arrows in Figures 6J–M; Supplementary Figure S5H). Taken together with the downregulation of *efnb2a* expression in the first pouch of *pax1a* mutants at 40 hpf, this result suggests that Pax1a could control HM plate formation through Efnb2a.

### Pax1a regulates HM plate formation through EphrinB2a in the first pouch

**FIGURE 7:**
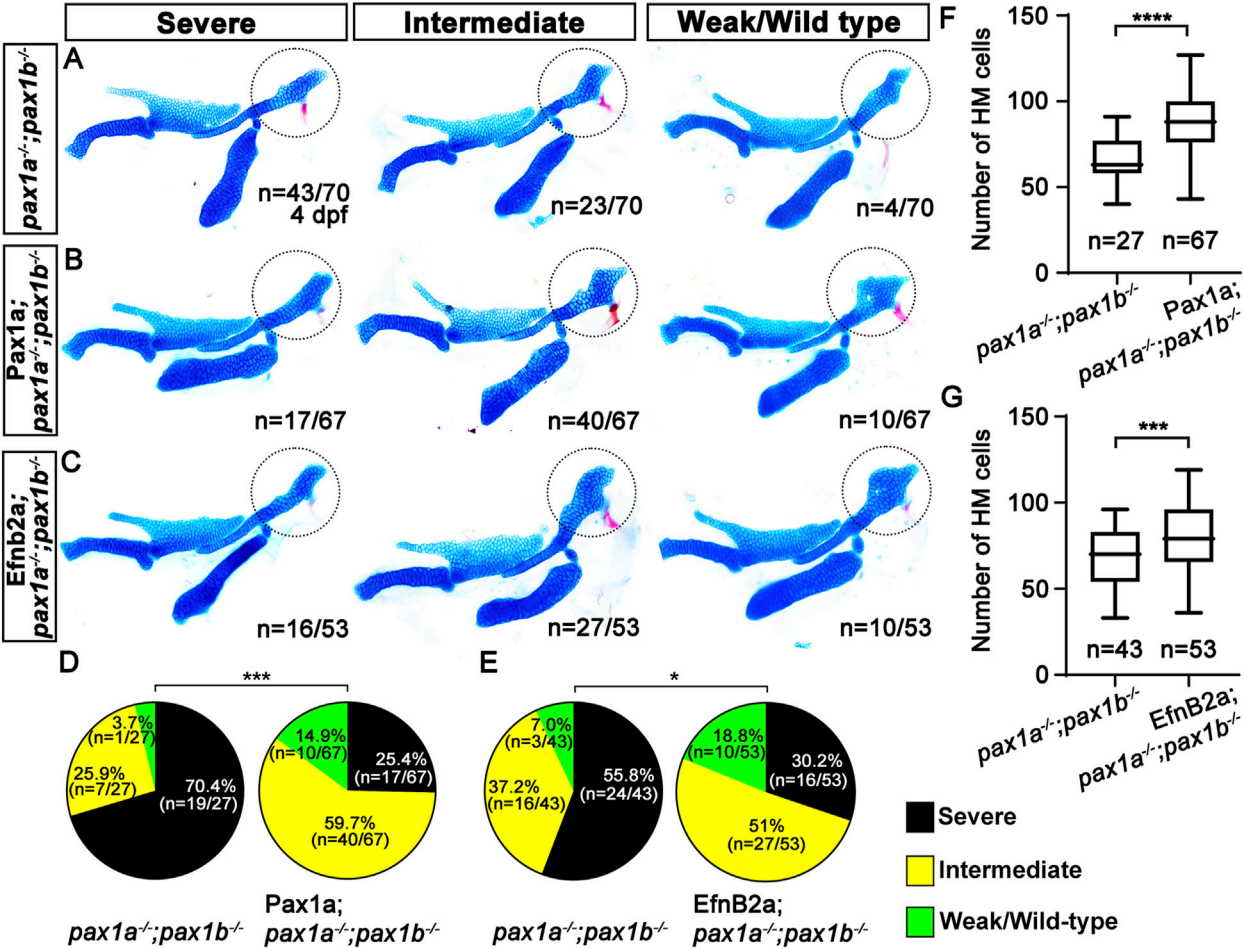
Requirement of Pax1a-EphrinB2a in the first pouch for hyomandibular plate development. **(A–C)** Unilateral dissections of the skeletons of the mandibular and hyoid arches stained with Alcian Blue (cartilage) and Alizarin Red (bone) at 4 dpf. HM plates are circled. The severe, intermediate, and weak defects in HM are seen in both *pax1* double mutants **(A)** and their siblings expressing Pax1a **(B)** or EfnB2a **(C)** in *sox17*-positive endoderm. Anterior is to the left. **(D, E)** Quantification of the frequency of each group between *pax1* double mutants and their siblings carrying *Tg(sox17:Gal4VP16)* and *Tg(UAS:Pax1a)* **(D)** or *Tg(sox17:Gal4VP16)* and *Tg(UAS:EfnB2a)* transgenes **(E)**. The frequency of each group is counted. Data is represented on a pie chart. Black, yellow, and green represent the severe, intermediate, and weak/wild-type groups. *** and * indicate *p* < 0.001 and *p* < 0.05, respectively. **(F, G)** Quantification of the number of cells in HM. The number of cells in HM is counted. Data is represented on a boxplot. **** and *** show *p* < 0.0001 and *p* < 0.001, respectively. n, number of animals analyzed.

To determine the genetic mechanism of Pax1a to control HM plate formation through EfnB2a, we tried to rescue the HM defects in the double mutants for *pax1a* and *pax1b* that showed complete penetrance of the phenotypes, either by spatially recovering *efnb2a* expression in the first pouch and by temporally inducing *efnb2a* expression at 39 hpf after pouch formation. We utilized the UAS/Gal4 system to induce EfnB2a expression in the pouches with the *Tg(sox17:Gal4VP16)* driver. The *Tg(sox17:Gal4VP16)* driver was able to induce expression of a target gene in the pouch endoderm, including the first pouch, at 36 and 48 hpf (Supplementary Figures S7A, B). While about 70.4 (n = 19/27) to 55.8% (n = 24/43) of double mutant siblings without transgenes displayed severe defects in the HM plate, with about 29.6 (n = 8/27) to 44.2% (n = 19/43) of animals showing intermediate or weak defects in the HM plate, about 25.4 (n = 17/67) to 30.2% (n = 16/53) of double mutant siblings expressing Pax1a or EfnB2a in *sox17*-positive endoderm showed severe defects in the HM plate, with about 74.6 (n = 50/67) to 69.8% (n = 37/53) of animals showing intermediate or weak defects in the HM plate (Figures 7A–C). The significant reduction in the severity of the defective HM plate in the double mutants supports the partial rescue by the induction of Pax1a or Efnb2a expression in *sox17*-positive endoderm in the double mutants (Figures 7D, E). The rescue was due to the increased number of cells composing the HM plate (Figures 7F, G; Supplementary Table S1). Importantly, this rescue effect was specific, as the induction of Pax1a or EfnB2a expression in the *sox17*-positive endoderm in wild types did not affect the development of facial skeletons (Supplementary Figures S7C–E). The partial rescue of the HM defects in the double mutants by the Efnb2a expression in the *sox17*-positive endoderm suggests that Efnb2a acts downstream of Pax1a in the first pouch for HM plate development.

**FIGURE 8:**
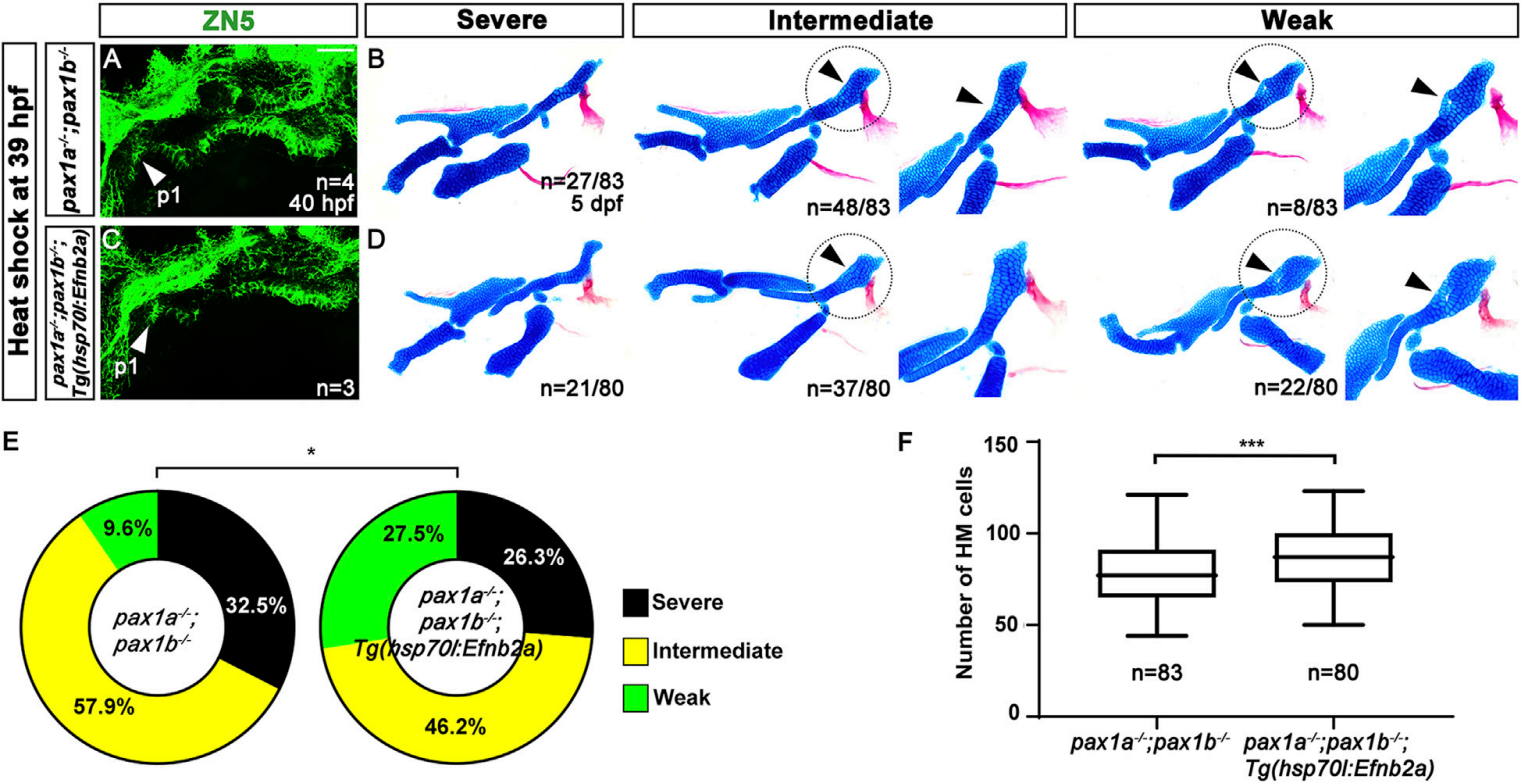
Requirement of Pax1a-EphrinB2a after pouch formation in hyomandibular plate development. **(A, C)** Zn5 immunohistochemistry to visualize pouches at 40 hpf. Arrowheads mark the first pouch (p1). After the heat-shock treatment at 39 hpf for 40 min, the first pouch is hypoplastic in *pax1* double mutants carrying *Tg(hsp70I:EfnB2a)* transgene, which is similar to the hypoplasia first pouch in *pax1* double mutants. Scale bar = 40 μM. **(B, D)** Lateral views of dissected facial skeletal elements of the mandibular and hyoid arches and inserts showing the zoomed-in HM plate (circled areas). After the heat shock treatment, the severe, intermediate, and weak defects in HM are seen in both *pax1* double mutants and those bearing *Tg(hsp70I:EfnB2a)* transgene. No wild-type HM plate is observed in **(B, D)**. Arrowheads indicate cells appearing in the HM plate in the intermediate group and the mono-layered cells adjacent to the foramen in the weak group. **(E)** Quantification of the frequency of each group between **(B, D)**. The frequency of each group is counted. Data is represented on a pie chart. Black, yellow, and green represent the severe, intermediate, and weak groups. * indicates *p* < 0.05. **(F)** Quantification of HM defects. The number of cells in HM is counted. Data is represented on a boxplot. *** indicates *p* < 0.001. n, number of animals analyzed.

Similarly, the *pax1*-dependent HM defects were partially rescued by inducing *efnb2a* expression after pouch formation with heat shock in the double mutants for *pax1a* and *pax1b* carrying *Tg(hsp70I:EfnB2a)* transgene (Figure 8). Compared to wild types, *efnb2a* expression in the pharyngeal region was significantly reduced in the double mutants for *pax1a* and *pax1b* at 40 hpf, with its expression not induced ectopically with heat-shock treatment at 39 hpf (Supplementary Figures S7G–I). However, heat-shock treatment at 39 hpf induced ectopic *efnb2a* expression in most areas of the double mutants for *pax1a* and *pax1b* carrying *Tg(hsp70I:EfnB2a)* transgene at 40 hpf (Supplementary Figure S7J), but with the first pouch still being malformed or hypoplastic (Figures 8A, C). We also verified that heat-shock treatment at 39 hpf in wild types bearing *Tg(hsp70I:EfnB2a)* transgene did not affect the facial skeletons at 5 dpf (Supplementary Figure S7F). After heat-shocking at 39 hpf, about 9.6% (n = 8/83) of double mutant siblings bearing no *Tg(hsp70I:EfnB2a)* transgene displayed weak defects in the HM plate, with about 90.4% (n = 75/83) animals showing intermediate or severe defects in the HM plate (Figure 8B). In contrast, after the heat-shock treatment, about 27.5% (n = 22/80) of double mutant siblings bearing *Tg(hsp70I:EfnB2a)* transgene showed weak defects in the HM plate, and about 72.5% (n = 58/80) animals showed intermediate or severe defects in the HM plate (Figure 8D). The increase in the degree of the weakly defective HM plate in the double mutants by the heat shock at 39 hpf was statistically significant (Figure 8E). In addition, after the heat shock, the number of cells composing the HM plate increased in the double mutants carrying *Tg(hsp70I:EfnB2a)* transgene compared to the double mutant siblings carrying no transgene (Figure 8F). The partial rescue of the HM defects in the double mutants by the Efnb2a expression after pouch formation suggests that EfnB2a acts downstream of Pax1a in HM plate development after pouch formation. Based on the rescues of *pax1*-dependent defects in the HM plate either by spatial or temporal recovering *efnb2a* expression, we propose that Pax1a controls HM plate development through Efnb2a in the first pouch.

## Discussion

In this study, we report a previously unappreciated role of Pax1a in the development of the HM plate. Although it was reported that Pax1a is entirely redundant with Pax1b in the morphogenesis of pouches that are essential for the development of facial cartilages (Liu et al., 2020), we found a role of Pax1a distinct from Pax1b in the first pouch formation as well as an independent role of Pax1a in the HM plate development, which is different from its role in the pouch formation. At the cellular levels, Pax1a is required to survive osteochondral progenitors differentiating into the chondrocytes in the hyoid arch during the HM plate development. Genetically, Pax1a controls the HM plate formation, in part, through Efnb2a. Here, we propose a model for the role of Pax1a controlling the HM plate development and discuss the distinct and redundant roles of Pax1a and Pax1b in facial cartilage development, as well as the broader significance of Pax1 in vertebrate jaw evolution.

### A model for the role of Pax1a in HM plate development

**FIGURE 9:**
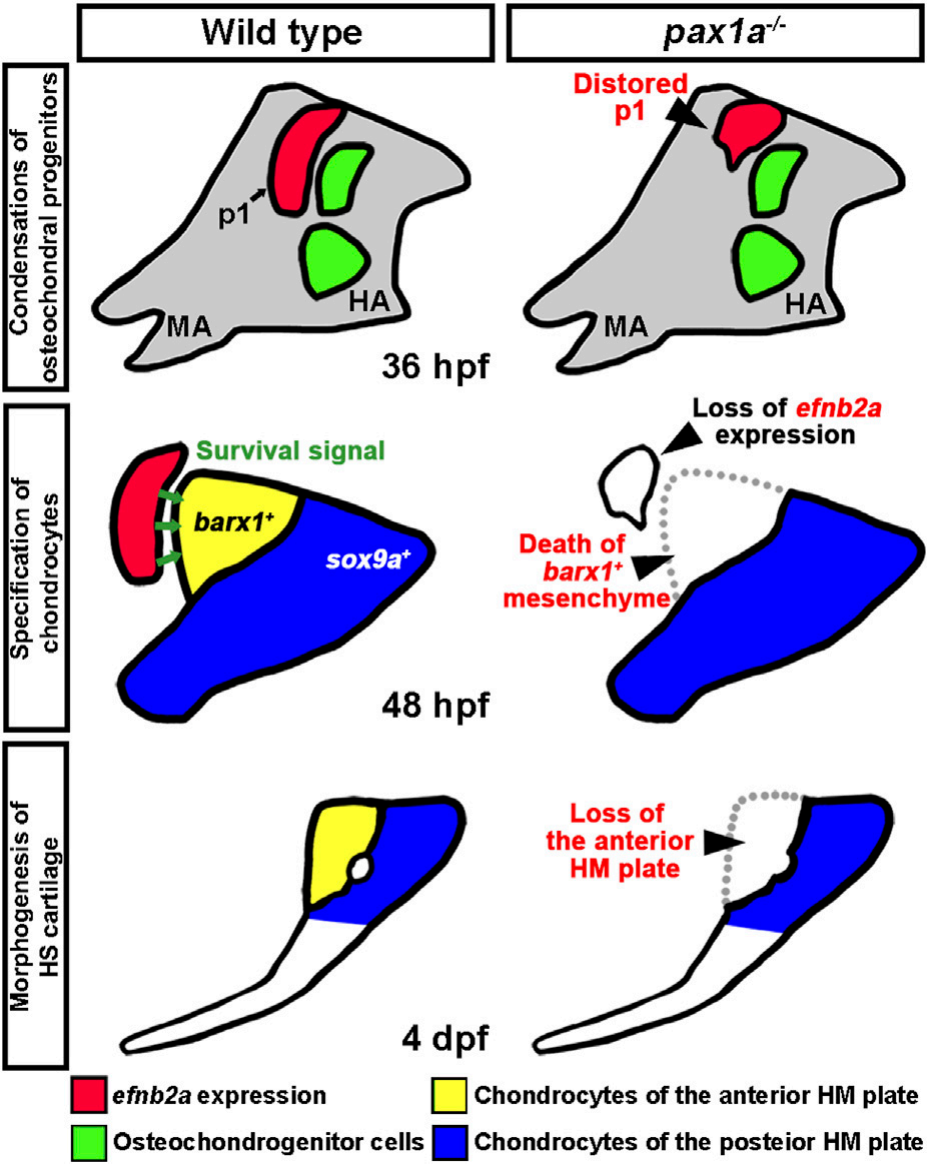
A model for the role of Pax1a in hyomandibular plate development. In wild types, Pax1a is necessary for the morphogenesis of the first pouch that forms at 18 hpf, with the loss of Pax1a leading to a distorted first pouch. After pouch formation, *pax1a* expression in the first pouch continues by 48 hpf, which is required to maintain *efnb2a* expression there. After the condensation of the skeletogenic mesenchyme in the hyoid arch at 36 hpf, Efnb2a in the first pouch would provide a survival signal to the *barx1*-positive osteochondral progenitors in the dorsal region of the hyoid arch that differentiate into chondrocytes contributing to the anterior plate of hyomandibular cartilage at 48 hpf. In *pax1a* mutants, reduced expression of *efnb2a* in the first pouch at 40 hpf would be insufficient to promote the survival of *barx1*-positive osteochondral progenitors in the dorsal region of the hyomandibular arch. Consequently, in *pax1a* mutants, osteochondral progenitors contributing to the anterior plate of hyomandibular cartilage die, and the anterior HM plate is defective in the larval head. p1, first pouch; MA, mandibular arch; HA, hyoid arch; HS, hyosymplectic cartilage; HM, hyomandibular cartilage.

Our work supports the role of Pax1a in the HM plate development as well as its role in the first pouch formation. Although the HM plate development largely relies on the first pouch (Crump et al., 2004b), our rescue analysis in the double mutants for *pax1* with the heat-shock treatment at 16 and 36 hpf supports the late role of Pax1a in the HM plate development along with its early role in the first pouch formation. Considering the expression patterns of *pax1a* and *pax1b* in the pharyngeal endoderm throughout the morphogenesis of pouches (Liu et al., 2020), the early expression of *pax1a* in the pharyngeal endoderm would be required for all pouch formation, including the first pouch, but with the first pouch formation requiring a Pax1a function distinct from Pax1b partially (Figure 9); in the *pax1a* mutants, the first pouch is distorted, with the other pouches being normal due to a high degree of genetic redundancy with *pax1b* (Figure 9). The continuous expression of *pax1a* in the mature first pouch adjacent to the dorsal region of the hyoid arch at 36 and 48 hpf would promote the survival of osteochondral progenitors differentiating into the chondrocytes that contribute to the HM plate, probably through the *efnb2a* expression in the first pouch (Figure 9); in the single mutants for *pax1a* and *efnb2a*, the differentiating *barx1*-positive osteochondral progenitors in the dorsal region of the hyoid arch adjacent to the mature first pouch could not receive a survival signal from the first pouch through the Pax1a-Efnb2a pathway, which would result in the loss of the chondrocytes contributing to the anterior part of the HM plate, consequently causing defects in the anterior part of the HM plate (Figure 9). The model for the role of Pax1a helps us better understand the genetic and cellular mechanisms by which this transcription factor orchestrates multiple stages of facial skeletal formation. Furthermore, although the Pax1a-Efnb2a pathway promoting cell survival has not been reported in the development of other tissues and organs, both Pax1 and EphrinB signaling have been implicated in the survival of thymic epithelial cells during embryonic development of the thymus in mice, with the epistasis between them undetermined, yet (Su et al., 2001; García-Ceca et al., 2013). Given that the thymus develops from the endoderm of the third pouch, it is likely that the Pax1a-Efnb2a pathway is recurrently used in the pouches for their developmental roles in the facial skeletons as well as in the glands forming from the pouches, including the thymus and parathyroid.

### Distinct and redundant roles of Pax1a and Pax1b in the development of facial cartilages

Similar to previous studies on the genetic compensations in mutants generated with the CRISPR/Cas9 system (Rossi et al., 2015; El-Brolosy et al., 2019), in the single mutants for *pax1a* and *pax1b*, we observed a genetic compensation in which the transcriptions of *pax1b* increase in *pax1a* mutants and *vice versa*, compared to wild types. Despite the genetic compensation, the defects in the first pouch and HM plate in *pax1a* mutants suggest that the increased transcription of *pax1b* is not able to compensate entirely for the role of Pax1a in the development of the first pouch and HM plate. In contrast, the normal development of the facial cartilages in *pax1b* mutants suggests that the increased expression of *pax1a* can replace the role of Pax1b. Moreover, the downregulation of *efnb2a* in the first pouch at 40 hpf in *pax1a* mutants in which *pax1b* expression increases suggests that the molecular function of Pax1a and Pax1b to regulate *efnb2a* expression would be different. Thus, Pax1a seems to have a distinct role from Pax1b in the first pouch and HM plate development. Alternatively, although Pax1a and Pax1b are redundant to each other in the first pouch and HM plate development, as previously reported (Liu et al., 2020), about a 1.5-fold increase in *pax1b* expression in *pax1a* mutants might not be sufficient to compensate for the role of Pax1a. In contrast, about a 12-fold increase in *pax1a* expression in the *pax1b* mutant might be enough to replace the role of Pax1b. Indeed, the increased severity and genetic penetrance of the HM defect in double mutants for *pax1a* and *pax1b*, in which both transcripts are significantly reduced, indicate redundant roles for these genes in developing the first pouch and HM plate. To determine the distinct role of Pax1a from Pax1b in the development of the first pouch and HM plate, it is necessary to analyze whether the defects in the double mutants for *pax1a* and *pax1b* can be rescued ultimately by recovering *pax1b* expression in the pharyngeal endoderm before the first pouch formation and after pouch formation. A positive result would support a redundant role of Pax1a and Pax1b, whereas a negative result would more likely support a distinct role of Pax1a from Pax1b in developing the first pouch and HM plate.

### Evolution of the role of Pax1 in craniofacial development

Our study provides an interesting scenario for the expanding role of Pax1 in craniofacial development during vertebrate jaw evolution. In hemichordates, gill slits first emerge, and the expression of *pax1/9* is associated with the development of these structures (Ogasawara et al., 1999). In prevertebrate chordates, a series of repetitive pharyngeal segments driven by endodermal pouches appear, and in jawless fish, these segmental structures give rise to cartilaginous gill baskets, with *pax1* and *pax9* expressed in the pouches, being associated with pouch formation and the segmentation of the pharynx (DeLaurier, 2019). Additional genes, such as *tbx1*, *fgf*s, and *efn*s, expressed in the pouches, were likely newly employed to form pouches in prevertebrate chordates or early vertebrate lineages (Jandzik et al., 2014; Koop et al., 2014). In the evolution of jawed vertebrates, these segments further developed into the complex architecture of the upper and lower jaws, as well as the supporting structures that keep them anchored (Donoghue et al., 2006), for which *pax1* and *pax9* that are initially involved in pouch formation, would have adapted to shape the skeletal elements in the adjacent arches by re-utilizing the signaling molecules, including the *efn* genes, that were utilized in pouch formation. Given that a general role of Fgf signal in specifying skeletogenic fate has been implicated in zebrafish through the expression of dominant-negative Fgf receptors and treatment with the FGFR inhibitor SU5402 (Walshe and Mason, 2003; Sperber and Dawid, 2008; Das and Crump, 2012), a straightforward way to test this scenario would be to examine the direct role of *fgf3*, which acts downstream of Pax1 in pouch formation in medaka and zebrafish, in the development of facial skeletal elements. Indeed, although the genetic interaction between Fgf3 and Pax1 has not yet been elucidated, we have recently found a role of Fgf3 in the development of epibranchial cartilages, a dorsal skeletal element that arises in the branchial arches, in zebrafish. Our analysis of the role of Pax1a reveals a previously unappreciated role in the development of HM plate and provides a novel insight into a genetic mechanism of the first pouch controlling the HM plate formation through the Pax1a-Efnb2a pathway, as well as its significance in the evolution of vertebrate jaws.

## Data Availability

The original contributions presented in the study are included in the article/Supplementary Material, further inquiries can be directed to the corresponding author.
