# Supplementary material for: Pax1a-EphrinB2a pathway in the first pharyngeal pouch controls hyomandibular plate formation by promoting chondrocyte formation in zebrafish
Source: Front Cell Dev Biol. 2025 Mar 5;13:1482906. doi: 10.3389/fcell.2025.1482906 (PMC11919851; doi:10.3389/fcell.2025.1482906)
Supplement: Supplementary file 1 [file DataSheet1.pdf]

SUPPLEMENTARY MATERIALS AND METHODS

Primers used to create transgenic constructs

Sox17-B4F: 5’-GGGGACAACTTTGTATAGAAAAGTTGCATTGTGTGCTTCTTGGTC-3’

Sox17-B1R: 5’-GGGGACTGCTTTTTTGTACAAACTTGTGAAACCTGAATGGCCTA-3’

Pax1a-B1F: 5’-GGGGACAAGTTTGTACAAAAAAGCAGGCTCCACCATGCTTTCGTGTTTTGCAG-3’

Pax1a-B2R: 5’-GGGGACCACTTTGTACAAGAAAGCTGGGTTGTCTATAACGGACGTGTCA-3’

Genotyping primers

pax1a-GT_F: 5’-TTGATTTAGGTCATGTGTGTTATATG-3’

pax1a-GT_R: 5’-TTTGTTTGTAGTCCCGTATGTTTTT-3’

pax1b-GT_F: 5’-GTTTTTCTGACAATGCAAAAAGTG-3’

pax1b-GT_R: 5’-CGTATTTCCCAAGCAAATATCC-3’

efnb2a-GT_F: 5’-GCGACTCTTTGTGGAGATA-3’

efnb2a-GT_R: 5’-CTGAACATCTGTGACTGGAA-3’

Primers used to create in situ probes

pax1a-r-F: 5’-GCTTTCGTGTTTTGCAG-3’

pax1a-r-R: 5’-TGTCTATAACGGACGTGTCA-3’

pax1b-r-F: 5’-AATCGGTGGGAGTAAACC-3’

pax1b-r-R: 5’-CTTATCGATGCCGTTGAC -3’

barx1-r-F: 5’-AGTTTCCGGTGTCTCCTC-3’

barx1-r-R: 5’-CCTCTTGGTTTGCATCAG-3’

sox9a-r-F: 5’-AGCAGCGATGTTATCGAA-3’

sox9a -r-R: 5’-GGACTGTGGTTGGATTGA-3’

SUPPLEMENTARY FIGURES

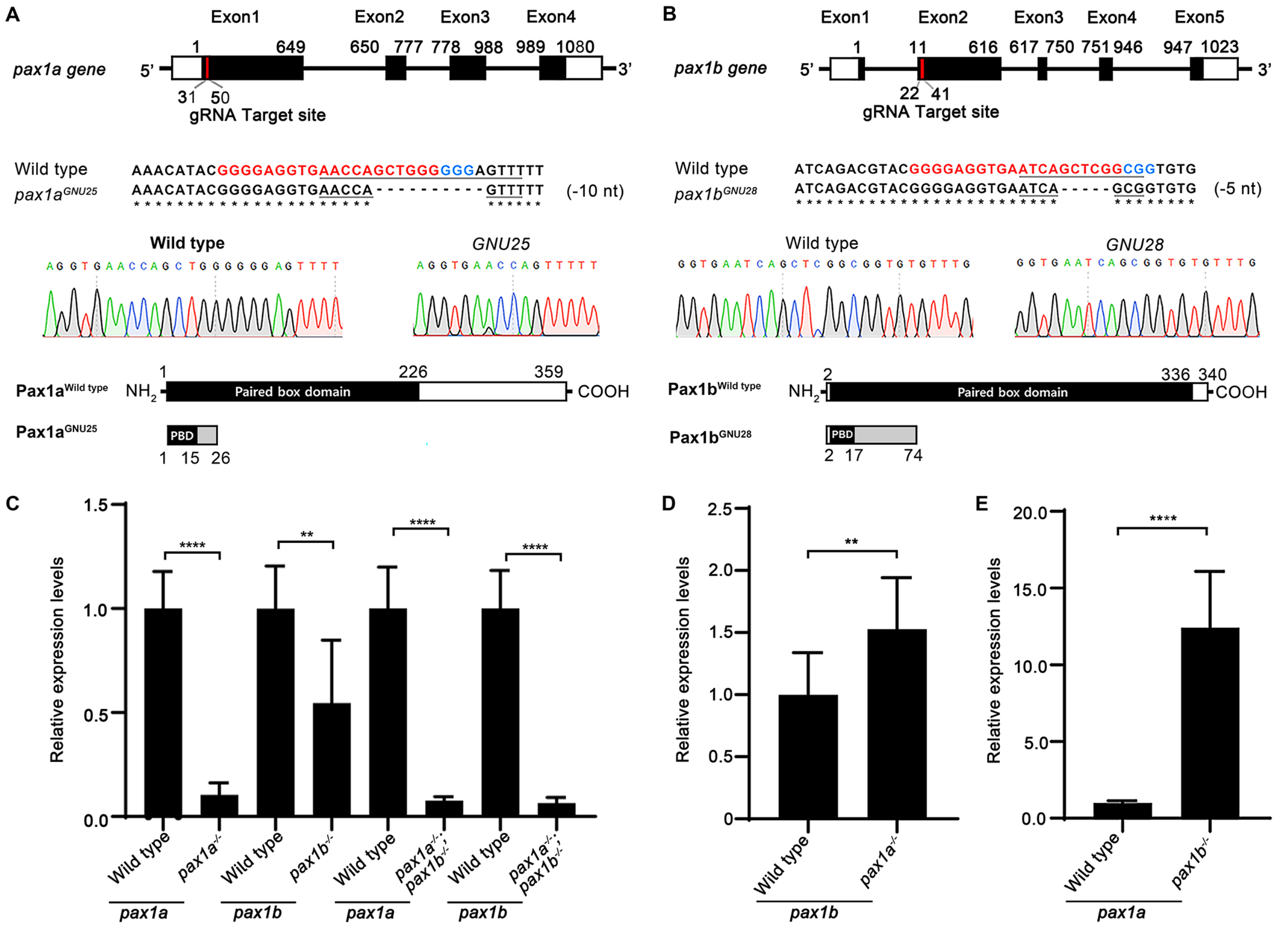

Supplementary Figure 1. Generation of loss-of-function alleles of *pax1* genes.

(A, B) The *pax1a* and *pax1b* genes contain four and five exons bearing sequences for the protein-coding region (black box) and the 5' and 3' untranslated regions (open box). The gRNA target site is marked in red. The deletion mutation of each mutant allele is shown in the multiple sequence alignments, with the gRNA target and the PAM sites being marked in red and blue, respectively, in the wild-type sequence. The electrophoretograms show the lesion in each mutant allele underlined in the multiple sequence alignments. Schematics of the Pax1a and Pax1b proteins encoded by the wild-type and mutant alleles show that most of the paired box domain (PBD) is missing due to an early truncation in the mutant Pax1a and Pax1b proteins.

(C-E) Relative expression levels of *pax1a* and *pax1b* mRNAs in wild types and single and double mutants of *pax1a* and *pax1b*. Expression in wild types set at 1. Data is represented on a column bar graph. Two and four asterisks show $p<0.01$ and $p<0.0001$.

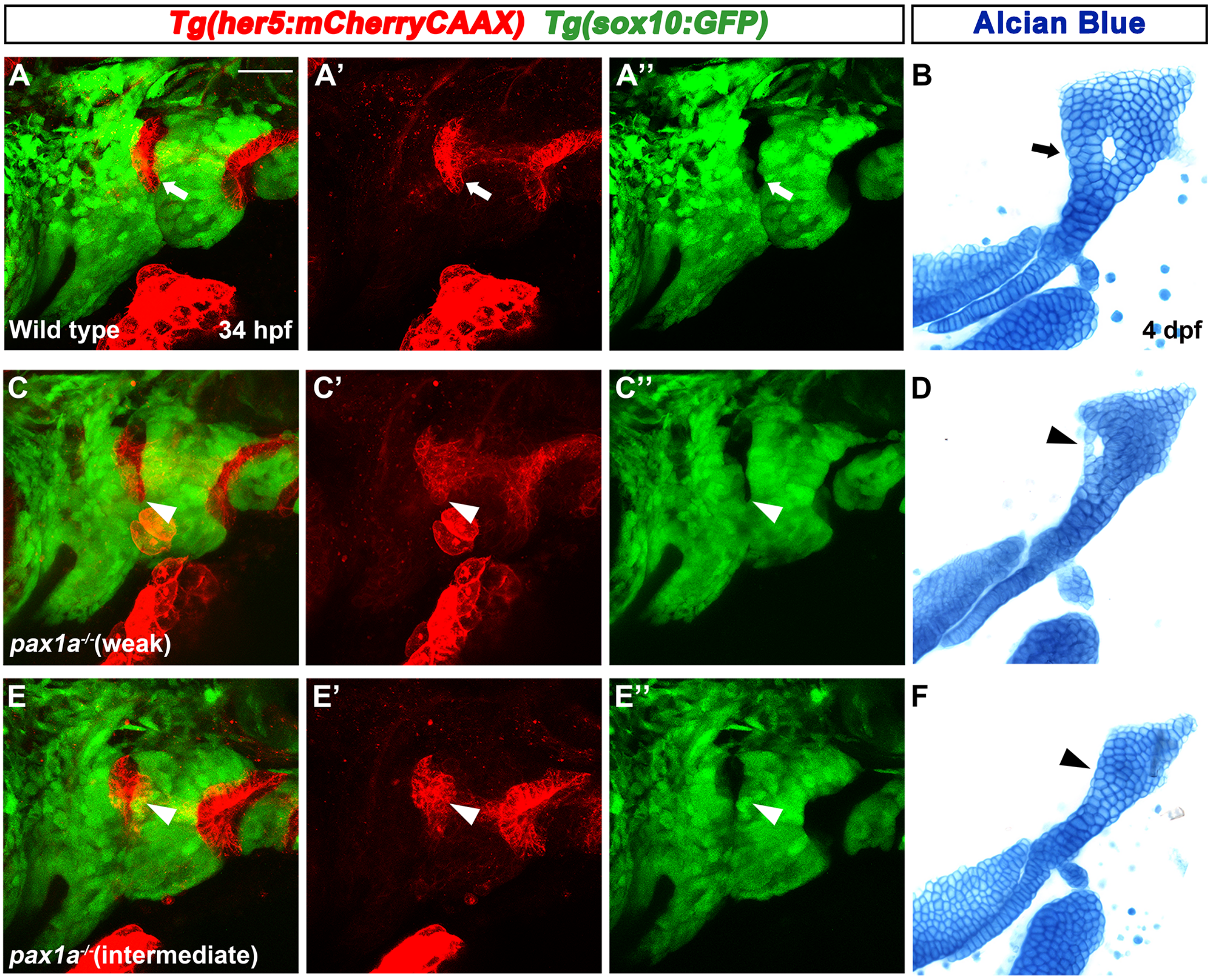

Supplementary Figure 2. Correlation of hyomandibular plate defects with earlier first pouch defects in the absence of Pax1a.

(A-F) Confocal projections from live imaging of embryos bearing *Tg(her5:mCherryCAAX)* (red) and *Tg(sox10:GFP)* (green) transgenes at 34 hpf, followed by Alcian Blue staining (blue) in the same individuals at 4 dpf.

(A, B) In a wild type, a bilayered normal first pouch is seen (white arrows), with normal HM observed later in the same animal (black arrow).

(C-F) Two examples of *pax1a* mutants developing a malformed first pouch earlier (white arrowheads) and an abnormal HM later (black arrowheads) in the same individual.

(A', C', E') Red channel only. **(A'', C'', E'')** Green channel only. Scale bar = 20 μM. Anterior is to the left.

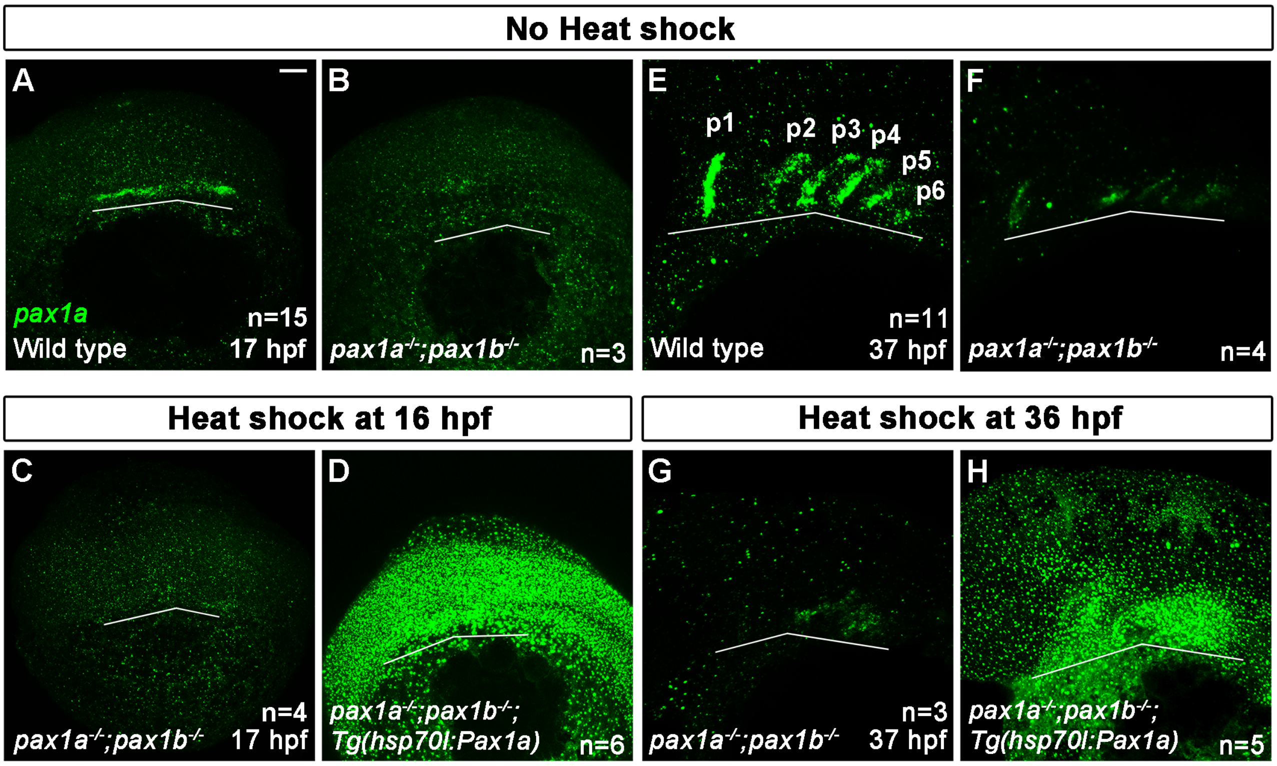

Supplementary Figure 3. Validation of the *Tg(hsp70l:Pax1a)* transgenic lines used to rescue *pax1a*-dependent hyomandibular plate defects.

(A-H) In situ hybridization for *pax1a* (green). The pharyngeal regions are underlined.

(A, B) While transcripts of *pax1a* are seen in the pharyngeal endoderm at 17 hpf before the first pouch forming in wild types, those are barely detected in *pax1* double mutant embryos, including the pharyngeal regions, at 17 hpf.

(C, D) Heat-shock treatment at 16 hpf for 40 minutes induces *pax1a* expression at 17 hpf in the entire areas of *pax1* double mutant embryos bearing *Tg(hsp70l:Pax1a)* transgenes, whereas it cannot in the sibling *pax1* double mutant embryos not carrying the transgenes.

(E, F) In wild types, transcripts of *pax1a* are detected in the pouches, including the first pouch at 37 hpf after pouch formation; those are hardly seen in *pax1* double mutant embryos at 37 hpf.

(G, H) While heat-shock treatment at 36 hpf for 40 minutes induces *pax1a* expression at 37 hpf in most areas of *pax1* double mutant embryos carrying *Tg(hsp70l:Pax1a)* transgenes, it cannot in the sibling *pax1* double mutant embryos not bearing the transgenes.

Scale bar = 10 μM. Anterior is to the left. n, number of animals analyzed.

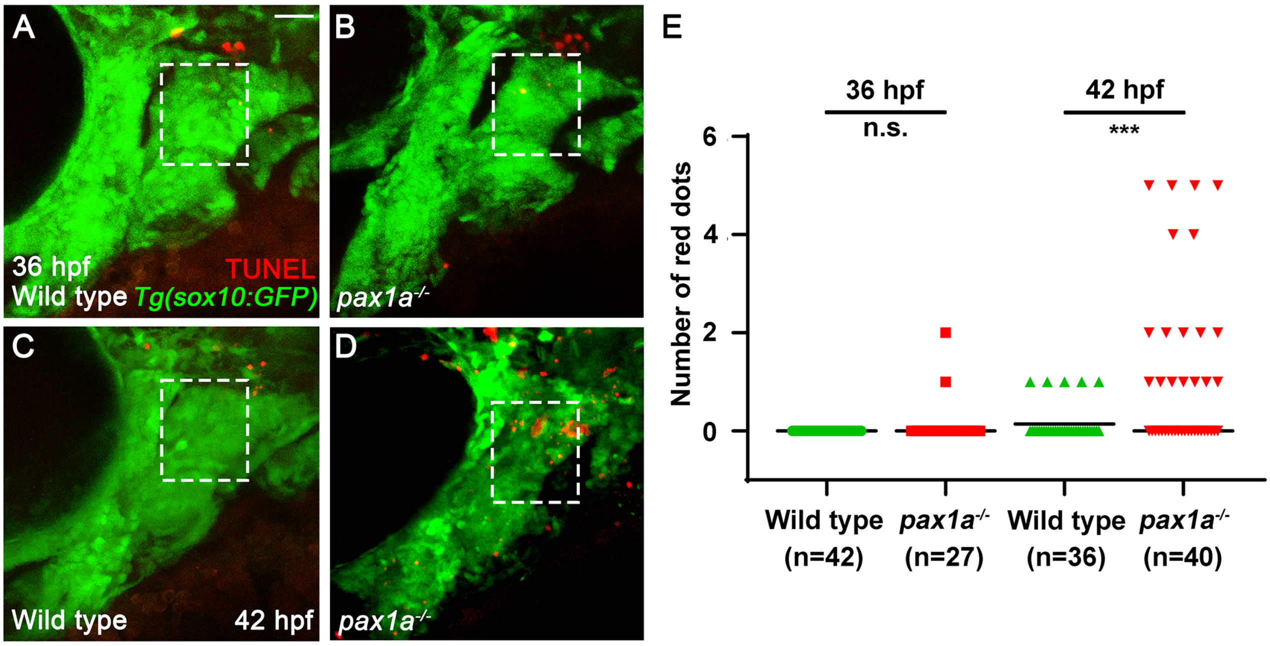

Supplementary Figure 4. Validation of the *pax1a*-dependent cell survival in the dorsal region of the hyoid arch.

(A-D) TUNEL staining (red) labels dying cells relative to *Tg(sox10:GFP)* expressing mesenchymal cells (green) in wild-type siblings and *pax1a* mutants. The dorsal regions of the hyoid arches are boxed. Scale bar = 20 μM. Anterior is to the left. Dorsal is at the top.

(E) Quantification of the number of dying cells in the dorsal region of the hyoid arch (boxed in A-D). Data is represented on a scatter plot. *** shows p<0.001. n.s., not significant.

n, number of animals analyzed.

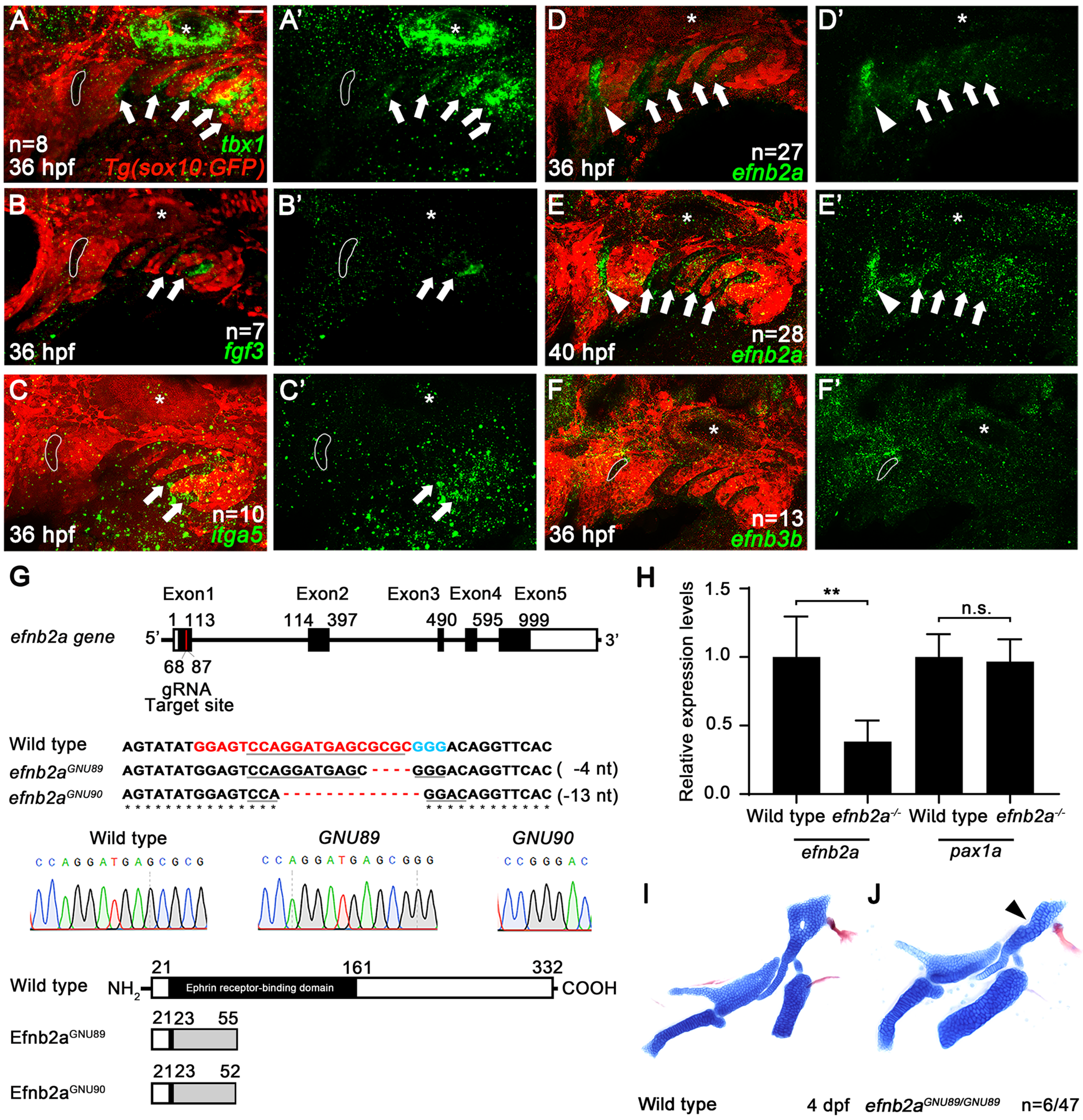

Supplementary Figure 5. Expression of the essential genes for the development of pouches after pouch formation, and generation of loss-of-function alleles of the *efnb2a* gene.

(A-F) In situ hybridization (green) in conjunction with the GFP immunohistochemistry (red) in wild types bearing *Tg(sox10:GFP)* transgene. **(A-C, F)** The first pouch is outlined.

(A) At 36 hpf, *tbx1* expression is seen in the second to sixth pouch (arrows) but not in the first pouch, with the expression in the anterior pouches gradually fading.

(B) At 36 hpf, *fgf3* is expressed in the fourth and fifth pouches (arrows), with the first, second, third, and sixth pouches not expressing it.

(C) Expression of *itg-α-5* is observed in the fifth and sixth pouches (arrows), with the other pouches not expressing it at 36 hpf.

(D, E) At 36 and 40 hpf, a relatively strong expression of *efnb2a* is observed in the first pouch (arrowheads), along with a fading expression of *efnb2a* seen in the other pouches (arrows).

(F) At 36 hpf, *efnb3b* expression is not seen in the pouches, including the first pouch.

(A'-F') Green channel only. Scale bar = 20 μM. Anterior is to the left. The ears are indicated with asterisks. n, number of animals analyzed.

(G) Generation of loss-of-function alleles of the *efnb2a* gene. The *efnb2a* gene consists of five exons bearing sequences for the protein-coding region (black box) and the 5' and 3' untranslated regions (open box). The gRNA target site is marked in red. The deletion mutation of each mutant allele is shown in the multiple sequence alignments, with the gRNA target and the PAM sites being colored in red and blue in the wild-type sequence. The lesion in each mutant allele underlined in the multiple sequence alignments is confirmed with the electrophoretograms. Schematics of the Efnb2a proteins encoded by the wild-type and mutant alleles show that most or all of the Ephrin receptor-binding domain is missing due to an early truncation in the mutant Efnb2a proteins.

(H) Relative expression levels of *efnb2a* and *pax1a* mRNAs in wild types and *efnb2a* mutants. Expression in wild types set at 1. Data is represented on a column bar graph. ** shows p<0.01. n.s., not significant.

(I and J) Lateral views of dissected facial cartilages. Compared to wild types, a distorted HM plate is seen in *efnb2a* mutants (arrowhead in J).

n, number of animals analyzed.

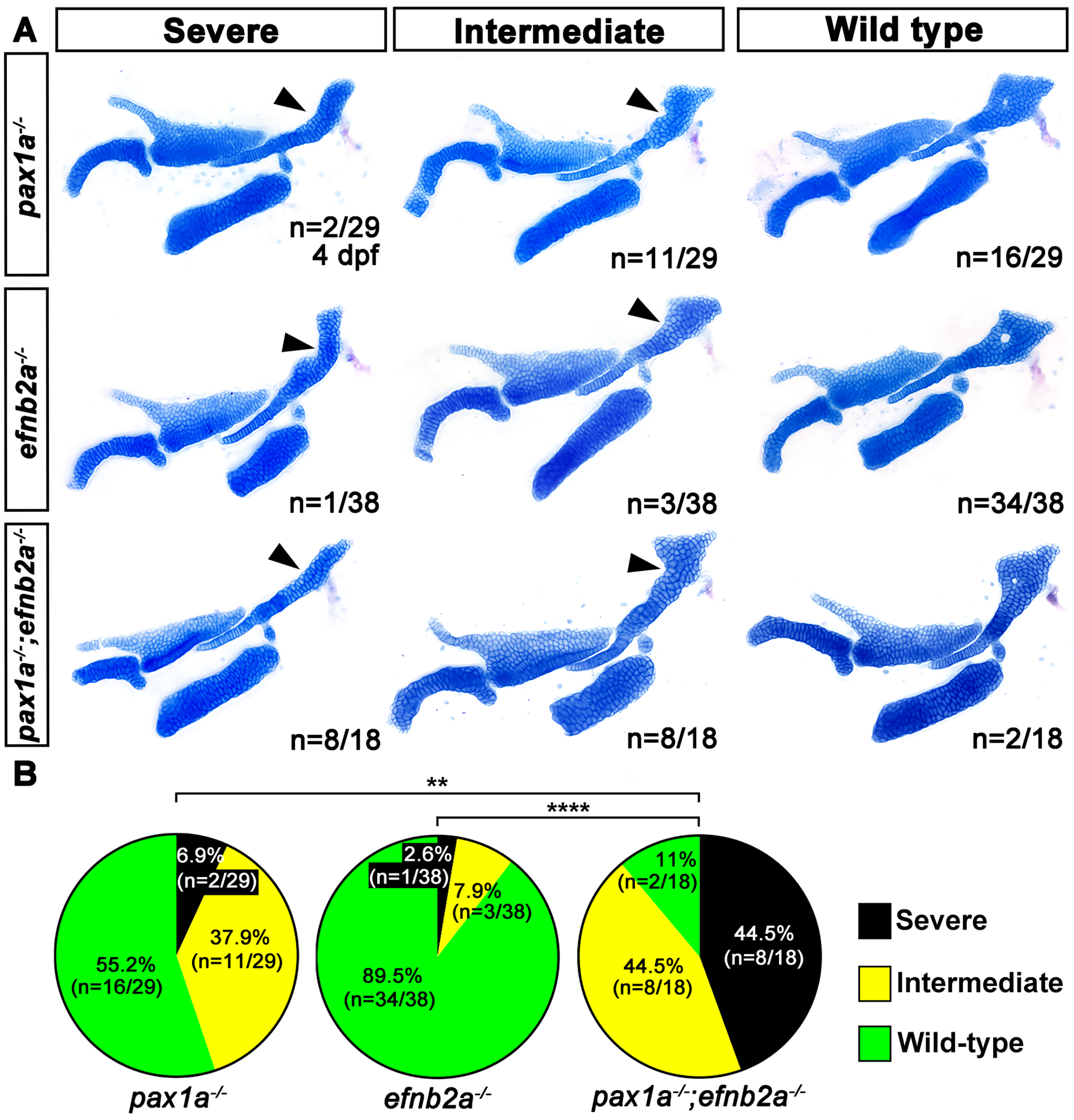

Supplementary Figure 6. Genetic interaction of EphrinB2a with Pax1a in hyomandibular plate development.

(A) Unilateral dissections of the skeletons of the mandibular and hyoid arches stained with Alcian Blue (cartilage) and Alizarin Red (bone) at 4 dpf. The severe and intermediate defects in HM are observed in single mutants for *pax1a* and *efnb2a* and double mutants for *pax1a* and *efnb2a*. Arrowheads indicate the defective HM plates. Anterior is to the left.

(B) Quantification of the frequency of each group among *pax1a* single mutants, *efnb2a* single mutants, and double mutants for *pax1a* and *efnb2a*. The frequency of each group is counted. Data is represented on a pie chart. Black, yellow, and green represent the severe, intermediate, and wild-type groups. **** and ** indicate $p<0.0001$ and $p<0.01$, respectively

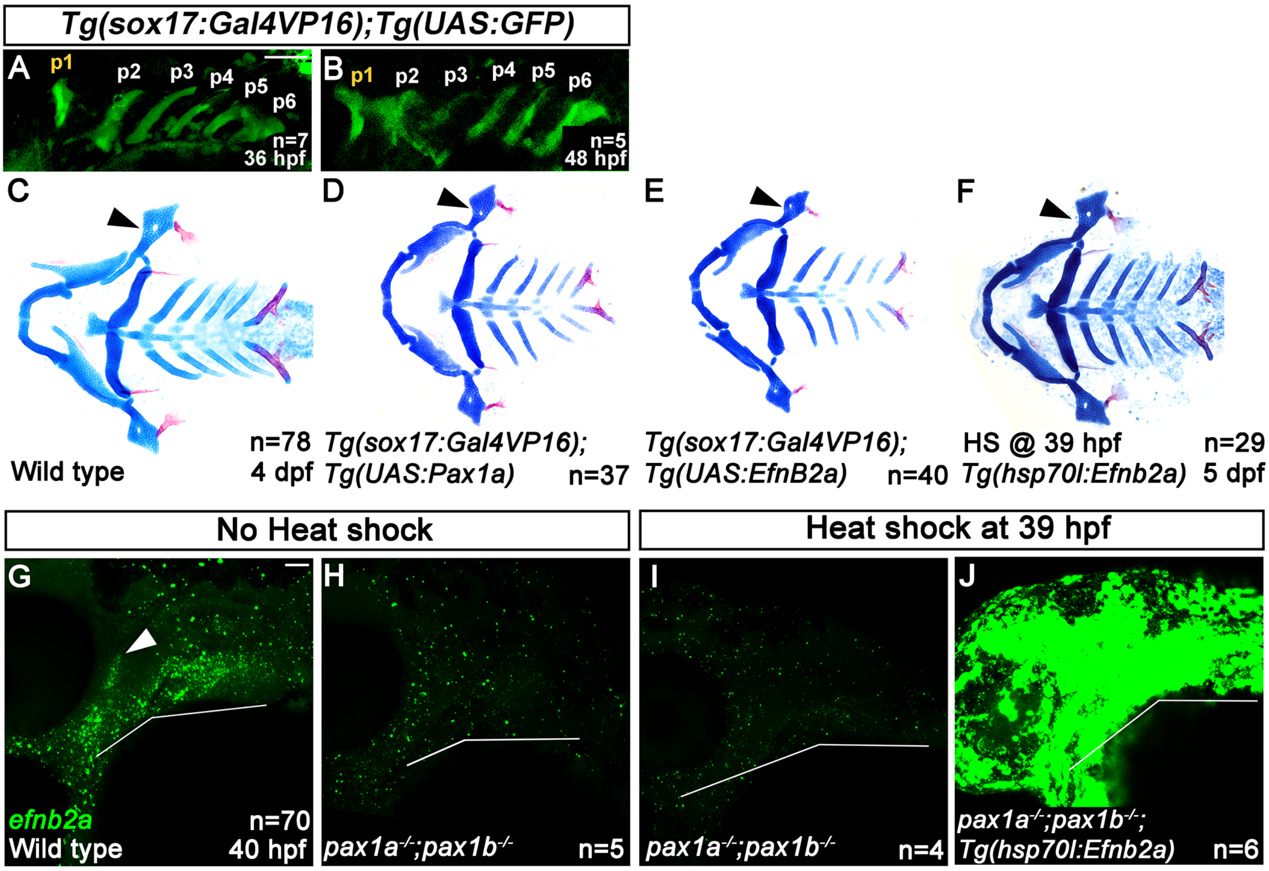

Supplementary Figure 7. Validation of the *Tg(sox17:Gal4VP16)* and *Tg(hsp70l:EfnB2a)* transgenic lines used to rescue *pax1a*-dependent hyomandibular plate defects.
(A, B) Confocal projections from live imaging of embryos bearing *Tg(sox17:Gal4VP16)* and *Tg(UAS:GFP)* transgenes. *Tg(sox17:Gal4VP16)* drives GFP expression in the pharyngeal endoderm and pouches, including the first pouch, at 36 and 48 hpf. The pouches are numbered. Scale bar = 40 μM. Anterior is to the left.

(C-F) Ventral views of dissected facial skeletons stained with Alcian Blue (cartilage) and Alizarin Red (bone). Compared to wild types, a forced expression of Pax1a or Efnb2a in the *sox17*-positive endoderm and pouches in wild-type animals leads to normal development of the facial skeletons, including the HM plate, at 4 dpf (arrowheads in C-E). Heat-shock treatment at 39 hpf in *Tg(hsp70l:Efnb2a)* transgenic animals shows normal facial skeletons, including the HM plate, at 5 dpf (arrowhead in F). HS, heat shock. Anterior is to the left.

(G-J) In situ hybridization for *efnb2a* (green). The pharyngeal regions are underlined. Scale bar = 10 μM. Anterior is to the left.

(G, H) In wild types, transcripts of *efnb2a* are detected in the pouches, including the first pouch (arrowhead) at 40 hpf after pouch formation; those are hardly seen in *pax1* double mutant embryos at 40 hpf.

(I, J) While heat-shock treatment at 39 hpf for 40 minutes induces *efnb2a* expression at 40 hpf in most areas of *pax1* double mutant embryos carrying *Tg(hsp70I:Pax1a)* transgenes, it cannot in the sibling *pax1* double mutant embryos not bearing the transgenes. Scale bar = 10 μM. Anterior is to the left.

n, number of animals analyzed.
